# The Peptide–Drug Conjugate M1pep–Tasquinimod Ameliorates Acute Pancreatitis via Selectively Clearing M1-like Macrophages

**DOI:** 10.34133/bmr.0250

**Published:** 2025-09-24

**Authors:** Fangyue Guo, Xufeng Tao, Zhiwen Zhai, Xin Kong, Yunfei Dai, Yu Wu, Yao Xu, Xinya Zhao, Jing Lv, Dong Shang, Hong Xiang

**Affiliations:** ^1^ Laboratory of Integrative Medicine, First Affiliated Hospital of Dalian Medical University, Dalian 116011, China.; ^2^ Department of Pharmacy, First Affiliated Hospital of Dalian Medical University, Dalian 116011, China.; ^3^ Centre for Animal Experiment, Wuhan University, Wuhan 430072, China.; ^4^ Department of Pharmacy, Dalian Medical University, Dalian 116044, China.; ^5^ Institute (College) of Integrative Medicine, Dalian Medical University, Dalian 116044, China.; ^6^ Department of General Surgery, First Affiliated Hospital of Dalian Medical University, Dalian 116011, China.

## Abstract

M1-like macrophages dominate local and systemic inflammatory response progression in acute pancreatitis (AP). The development of strategies to target pro-inflammatory M1-like macrophages in conjunction with primary pathophysiology-specific pharmacological therapy presents a challenge in the management of AP. Peptide–drug conjugates (PDCs), which are emerging second-generation conjugate drugs, have quickly become a new favorite in the field of targeted drug delivery due to their superior drug bioavailability, affinity, and stability. Tasquinimod (Tasq) is a specific inhibitor of S100A9 that is expressed mainly in M1-like macrophages during AP. Drug repositioning revealed that Tasq improved AP in a dose-dependent manner, but drug toxicity occurred at doses of 30 mg/kg. Therefore, we selected 2 specific M1-like macrophage-binding peptides (M1peps) by phage display technology and developed a novel PDC, M1pep-Tasq, by connecting M1peps to activated Tasq with a cleavable linker. Based on a mouse model of AP constructed by retrograde injection of sodium taurine cholate into the bile pancreatic duct and an M1-like macrophage polarization model induced by lipopolysaccharide + interferon-γ stimulation, we confirmed that M1pep-Tasq reduces the drug toxicity of Tasq and improves its efficacy by enhancing the targeting of Tasq to damaged organs in vivo and to M1-like macrophages in vitro. Furthermore, M1pep-Tasq effectively improves AP by inhibiting M1-like macrophage polarization by suppressing the S100A9–TLR4–MAPK pathway. Overall, we have developed a novel PDC, M1pep-Tasq, with promising applications in clinical settings to treat a range of inflammatory disorders by increasing the efficacy and reducing the toxicity of Tasq.

## Introduction

Acute pancreatitis (AP) is an inflammatory disorder of the exocrine pancreas associated with tissue injury and necrosis. The incidence of AP has increased by more than 2% to 5% per year and varies between 3.4 and 73.4 cases per 100,000 worldwide [1]. Although most patients (up to 70%) with AP have a mild and self-limiting disease course, approximately 1 in 3 patients experiences moderate or severe AP, with a risk of death as high as 30% to 40% [2]. Among the cell types involved in pancreatitis, macrophages play a central role in coordination. When exposed to lipopolysaccharide (LPS), interferon-α, IL-12, IL-23, and other factors, resting macrophages (M0) are polarized into pro-inflammatory M1-like macrophages (M1), leading to local or systemic inflammatory responses [3]. Research on the involvement of macrophages in AP is burgeoning [4]. Nevertheless, there are few effective treatments that can reverse the polarization of M1-like macrophages, which makes the search for new therapeutic avenues critically important.

The calcium-binding protein S100A9, also known as myeloid-related protein 14, is derived primarily from monocyte-macrophages [5]. In AP, S100A9 participates in early inflammation and tissue injury by stimulating pro-inflammatory cytokine expression [6]. Moreover, the up-regulation of S100A9 aggravates LPS-induced acute lung injury by activating macrophage M1 polarization and pyroptosis via the TLR4/MyD88/NF-κB pathway, which suggests a potential key target for AP therapy [5]. Tasquinimod (a quinoline-3-carboxyamide, Tasq), as a small-molecule immunotherapy targeting S100A9, has been shown to affect the accumulation and function of suppressive myeloid cell subsets in tumors; its outstanding immunomodulatory effects also indicate its potential for treating inflammatory diseases [7]. Although Tasq has the potential to prolong progression-free survival in phase II clinical trials, it has failed to meet the critical endpoint of overall survival in phase III clinical trials [8–10]. The presence of adverse reactions, especially serious adverse reactions that may lead to treatment discontinuation (such as deep vein thrombosis and cardiovascular events), has limited its widespread clinical use. Efforts to repurpose Tasq for rare diseases (such as multiple myeloma) through the Food and Drug Administration’s (FDA’s) “orphan drug” designation (from 2017 to 2022) have encountered the same safety barriers in ongoing trials [11]. Despite the use of dose-adjustment strategies, dose-limiting toxicities have persisted. Overall, the results of the clinical trials of Tasq indicate that although it has some effect on treating inflammation, adverse reactions have limited its further development and market approval. Future efforts may need to further optimize drug formulations or identify safer usage protocols to overcome these challenges.

Drug-targeting strategies offer new opportunities for repurposing drugs that have failed in clinical trials. Following the success of antibody–drug conjugates, peptide–drug conjugates (PDCs) have emerged as a rising star in targeted drug delivery [12]. PDCs, which are composed of a drug, a cleavable linker, and a multifunctional peptide, enable precise drug delivery while minimizing adverse effects [13,14]. Despite the broad applications of PDCs, research targeting macrophages—especially M1 macrophages—remains scarce and is currently unexplored.

**Fig. 1.**
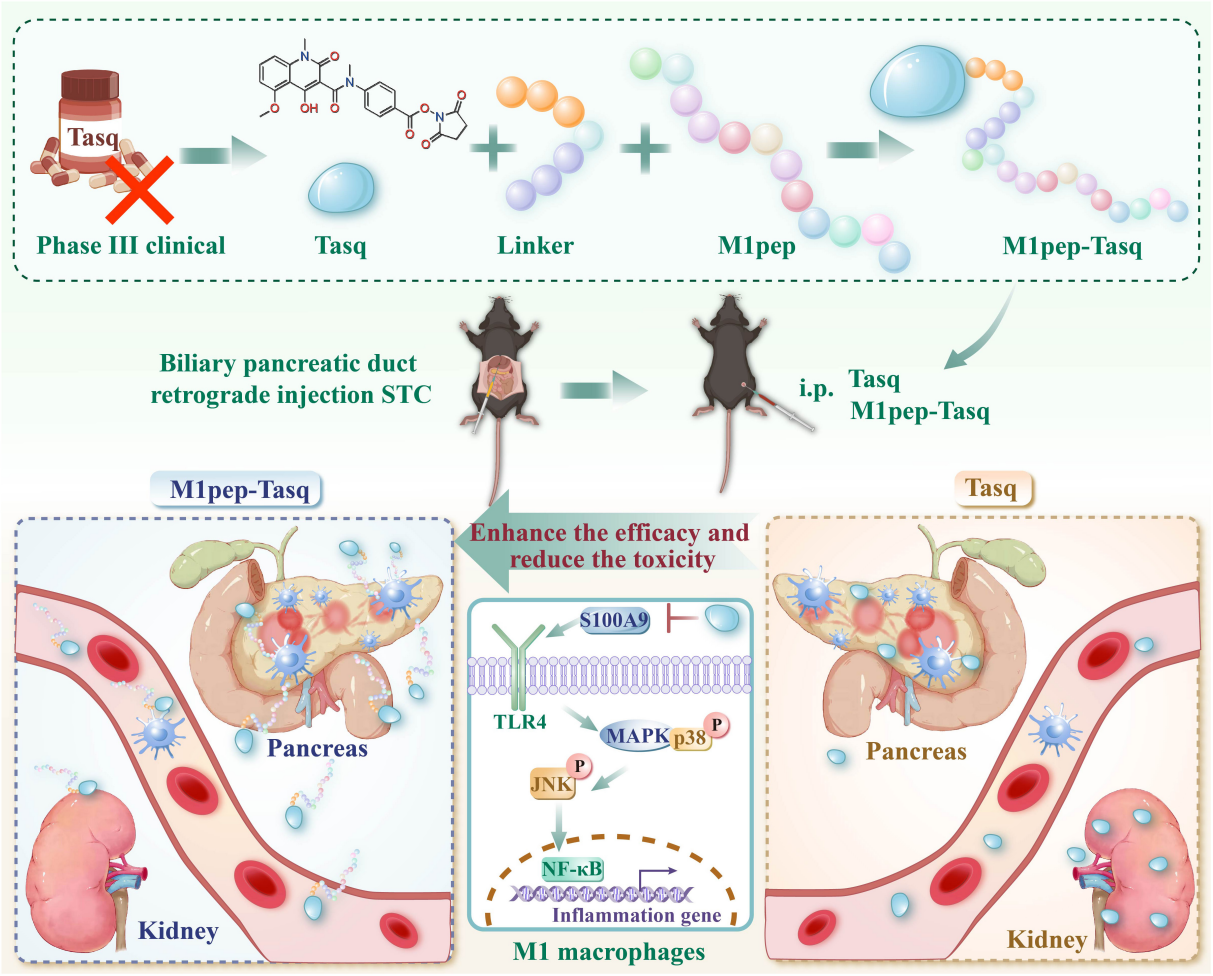
The novel PDC drug, M1pep-Tasq, was synthesized by conjugating M1pep to activated Tasq via a flexible linker. M1pep-Tasq increases the targeting of Tasq to M1-like macrophages, significantly improving the efficacy of Tasq in treating AP and reducing its renal toxicity. The mechanisms underlying the reduced toxicity and enhanced efficacy of M1pep-Tasq may involve inhibiting the S100A9-TLR4- MAPK pathway to suppress the polarization of M1-like macrophages.

In this study, we utilized phage display technology to identify peptides (macrophage-binding peptides [M1peps]) that target M1-like macrophages and synthesized a PDC (M1pep-Tasq) by coupling M1pep with Tasq to increase the efficacy of Tasq and reduce its adverse effects (Fig. 1).

## Materials and Methods

### Cell culture

The RAW264.7 cell line and THP-1 cell line were purchased from the American Type Culture Collection (VA, USA). RAW264.7 cells were cultured in high-glucose Dulbecco’s modified Eagle’s medium (DMEM) supplemented with 10% fetal bovine serum (FBS). THP-1 cells were cultured in RPMI 1640 medium supplemented with 10% FBS and 0.05 mM β-mercaptoethanol. The cells were incubated under 5% CO_2_ at 37 °C. All cell lines were authenticated by short tandem repeat profiling and were regularly tested for mycoplasma contamination. For all experiments, THP-1 cells were cultured in 6-well plates and treated with 100 nM phorbol 12-myristate 13-acetate for 48 h to transform into adherent macrophages.

### Phage display technique

To induce M1-like macrophages, RAW264.7 cells were stimulated with 1 μg/ml LPS + 20 ng/ml interferon-γ (IFN-γ) for 24 h. The cells were then incubated with phage display libraries (heptapeptide and dodecamer libraries) at room temperature with rotation, followed by elution and titration. After 4 to 5 rounds of this process, flow cytometry validation was performed, and positive clones were selected, analyzed, and sequenced.

### PDC synthesis

We conjugated the structurally modified Tasq with a peptide. First, for the synthesis and modification of Tasq via solid-phase peptide synthesis, we activated the resin with dichloromethane. We then coupled the first amino acid in the presence of diisopropylethylamine and dimethylformamide, followed by extensive washing. Deprotection was performed using a solution of piperidine in dimethylformamide, and success was confirmed with a ninhydrin test, where a blue color indicated a positive reaction. The resin was washed again, and the next amino acid was coupled using *O*-(7-azabenzotriazol-1-yl)-*N*,*N*,*N*′,*N*′-tetramethyluronium hexafluorophosphate. This cycle of coupling, deprotection, and testing was repeated for each subsequent amino acid. Once the peptide chain was fully elongated, the resin was washed, and the peptide was cleaved off using a trifluoroacetic acid-based solution. The crude peptide was then purified through precipitation with diethyl ether, yielding the final product.

### Animals

Wild-type male C57BL/6 mice (20 to 22 g) were provided by the Experimental Animal Centre of Dalian Medical University, and all animal care and experimental procedures were approved by the Dalian Medical University Animal Care and Use Committee.

### Isolation and culture of bone-marrow-derived macrophages from mice

After the 6- to 8-week-old mice were anesthetized and blood was collected, they were soaked in 75% alcohol for 2 to 3 min. Under a laminar flow hood, the hind limbs were cut off, the muscles were removed, and the tibiae and femurs were removed. The tibiae and femurs were cut in half from the middle. The marrow cavity was rinsed with phosphate-buffered saline (PBS) until the bone was translucent, and a cell suspension was obtained. The cell suspension was filtered through a 200-mesh stainless steel cell sieve. The cell suspension was collected, and 1,000 μl of red blood cell lysis solution was added. The sample was washed twice with 4 ml of PBS. The cells were resuspended in DMEM containing granulocyte-macrophage colony-stimulating factor and transferred to a primary cell incubator for culture at 37 °C with 5% CO_2_. The medium was changed on day 3 of culture. By day 5, the adherent cells were identified as bone-marrow-derived macrophages (BMDMs). The cells were incubated with 1 μg/ml LPS and 20 ng/ml IFN-γ for 24 h to induce the differentiation of BMDMs into M1-type macrophages. The cells were incubated with 20 ng/ml IL-4 for 48 h to induce the differentiation of BMDMs into M2-type macrophages.

### Extraction of immune cells from the peripheral blood of mice

The mice were intraperitoneally injected with 10 mg/kg LPS, and 24 h later, the peripheral blood of each mouse was collected. A mouse peripheral blood neutrophil isolation kit (Solarbio, Beijing, China) was used to extract immune cells from the peripheral blood of the mice. Following the kit’s instructions, fresh anticoagulated whole blood was diluted with 1× PBS at a 1:1 ratio. Then, a separation reagent was added, and the mixture was centrifuged at 1,000 × g using a horizontal rotor at room temperature for 30 min to obtain mouse peripheral blood neutrophils and mononuclear cells. The cells were incubated in RPMI 1640 medium supplemented with 10% FBS and 1% penicillin–streptomycin.

### In vivo toxicity

To assess the toxicological effects of Tasq, C57BL/6 mice were allocated into control, Tasq-15-treated (Tasq at a dosage of 15 mg/kg), and Tasq-30-treated (Tasq at a dosage of 30 mg/kg) groups. Tasq was dissolved in a saline solution containing 5% dimethyl sulfoxide (DMSO) for oral administration. After the mice were euthanized, the serum, heart, liver, spleen, lungs, kidneys, intestines, and pancreas were harvested.

### In vivo efficacy evaluation

After anesthesia, a midline incision of approximately 1 cm was made at the mouse portal vein, and the common hepatic duct was temporarily clamped to prevent liver reflux. Then, under the guidance of a small-animal microscope (Olympus Corp, Tokyo, Japan), 3% sodium taurocholate (STC) or 0.9% sodium chloride (40 μl) was injected into the biliopancreatic duct for 5 min. Mice in the treatment groups were administered Tasq-15 (Tasq at a dosage of 15 mg/kg), Tasq-30 (Tasq at a dosage of 30 mg/kg), M1pep-Tasq-15 (Tasq at a dosage of 15 mg/kg), M1pep-Tasq-30 (Tasq at a dosage of 30 mg/kg), NCpep-Tasq-15 (Tasq at a dosage of 15 mg/kg), or NCpep-Tasq-30 (Tasq at a dosage of 30 mg/kg) via intraperitoneal injection immediately after modeling at 2 h. The mice were given an equal volume of 2% β-cyclodextrin + 5% DMSO at the same time point as the AP group. The mice were euthanized 24 h after Tasq administration, and the serum was collected, with the heart, liver, spleen, lungs, kidneys, intestines, and pancreas preserved in a 4% paraformaldehyde solution.

### Biochemical index detection

Serum levels of amylase (AMS), lipase (LIP), creatinine (CRE), blood urea nitrogen (BUN), alanine aminotransferase (ALT), and aspartate aminotransferase (AST) were determined using appropriate assay kits (Njccbio, Nanjing, China). More detailed steps can be found in the Supplementary Materials.

### C-reactive protein detection

The content of C-reactive protein (CRP) in mouse serum was detected according to the instructions of Mouse CRP ELISA Kit (Jonlnbio, Shanghai, China). Serum samples and standard solutions at varying concentrations were added to designated wells and incubated at 37 °C for 60 min. Biotinylated detection antibody working solution was then directly added to each well. After plate washing, enzyme conjugate working solution was added. Subsequently, a substrate (3,3′,5,5′-tetramethylbenzidine) was added to each well and incubated at 37 °C for 15 min under light-protected conditions. Finally, the reaction was terminated with stop solution, and the absorbance value was measured at 450 nm.

### Hematoxylin and eosin staining

Hematoxylin and eosin (HE) staining was used to detect pathological tissue damage in mice. The 4% paraformaldehyde-fixed pancreatic tissue was taken out, embedded in paraffin, and cut into tissue sections approximately 5 μm thick, and the sections were fixed on glass slides treated with polylysine. Subsequently, the paraffin sections were immersed in xylene and 100%, 95%, 85%, and 75% ethanol in sequence, followed by HE staining. Images of the tissue sections were obtained at a magnification of ×200 using a light microscope (Olympus Corp, Tokyo, Japan).

### Flow cytometry

Peptides conjugated with the fluorescein isothiocyanate (FITC) fluorescent group were dissolved in PBS buffer and diluted to a 10 μM working solution with culture medium before the experiment; RAW264.7 cells and THP-1 cells were induced with 100 ng/ml LPS and 20 ng/ml IFN-γ to become M1-type macrophages, and untreated RAW264.7 and THP-1 cells were used as negative controls. Primary BMDMs, dendritic cells (DCs), neutrophils, and T cells were extracted from mice, respectively, with a portion of each type of cell as a negative control. Both the negative and positive control groups were incubated with 1 ml of the peptide working solution for 4 h in the dark. A flow cytometer (BD, NJ, USA) was used to analyze the binding of the peptide to M1-type macrophages.

### Laser confocal microscopy

RAW264.7, THP-1, and mouse BMDM cells were seeded into a laser confocal culture dish, and 1 ml of the peptide working solution was added, followed by incubation in the dark for 4 h. The cells were fixed with a 4% paraformaldehyde solution for 20 min at room temperature. Phalloidin-iFluor 594 antibody (Abcam, Cambridge, UK) (1:1,000) was added, and the mixture was incubated in the dark for 2 h. 4′,6-Diamidino-2-phenylindole solution was then added, and the mixture was incubated for 30 min. A laser confocal microscope (Leica, Wetzlar, Germany) was used to analyze the binding of the peptide to M1-type macrophages.

### Real-time quantitative polymerase chain reaction

Total RNA was extracted from RAW264.7 and THP-1 cells subjected to different treatments using an RNAex Pro RNA reagent kit (Accurate Biology, Hunan, China). Reverse transcription was performed using an All-in-One First-Strand Synthesis MasterMix (with dsDNase) (Yugong Biolabs, Jiangsu, China) on a GeneExplorer polymerase chain reaction (PCR) system (Bioer, Zhejiang, China). Relative messenger RNA (mRNA) expression levels were quantified with Taq-HS SYBR Green qPCR Premix (Universal) (Yugong Biolabs, Jiangsu, China) on an ABI 7500 Real-Time PCR system (Applied Biosystems, Foster City, CA). The expression levels of all genes were normalized to the expression of β-actin, and the fold changes among different groups were calculated using the 2^−ΔΔCT^ method for quantitative analysis. The sequences of primers used in this study are listed in Table S1.

### Imaging of live small animals

A mouse model of AP was constructed using retrograde injection of STC. Two hours later, the mice were administered FITC-pep-Tasq or FITC-Tasq via tail vein injection. Small-animal in vivo imaging (PerkinElmer, MA, USA) was performed, and photos were obtained at 15 min, 30 min, and 1 h post-administration.

### Cell viability assay

The optimal working concentrations of Tasq, M1pep-Tasq, and NCpep-Tasq were determined using a cell viability assay. Tasq, M1pep-Tasq, and NCpep-Tasq were dissolved in DMSO and then added to the cells. The final concentration of DMSO was less than 0.1%. Briefly, RAW264.7 cells were seeded into a 96-well plate at a density of 1 × 10^5^ cells/ml, incubated for 24 h, and subsequently incubated with varying concentrations (100, 50, 20, 10, 5, 2, and 1 μM) of Tasq, M1pep-Tasq, or NCpep-Tasq at 37 °C in a 5% CO_2_ environment for 24 h. Then, 10 μl of Cell Counting Kit-8 solution was added to each well, and the cells were incubated for an additional hour. The absorbance at 450 nm was measured using a microplate reader (BioTek, Burlington, VT).

### RNA sequencing

RAW264.7 cells were induced with LPS + IFN-γ for 24 h, followed by treatment with Tasq and M1pep-Tasq for an additional 24 h. Total RNA was extracted using TRIzol reagent according to the manufacturer’s instructions, and genomic DNA was removed from the preparation using DNase I. Library preparation and sequencing were completed by Technology Co., Ltd. (Hangzhou, China) on an Illumina NovaSeq 6000 platform. DESeq2 (version 1.12.4) was used for the analysis of differentially expressed genes (DEGs). Genes with a *q* value (false-discovery-rate-adjusted *P* value) <0.05 and a fold change >1.5 were selected as the thresholds for marked differences.

### Western blot

Total protein was extracted from the cells, quantified using a bicinchoninic acid assay kit, and separated by sodium dodecyl sulfate–polyacrylamide gel electrophoresis on 10% to 12% gels. The proteins were then transferred onto polyvinylidene fluoride membranes, blocked with 5% skim milk, and incubated overnight at 4 °C with specific primary antibodies against the proteins of interest (S100A9, TLR4, p38 MAPK, p-p38 MAPK, JNK, and p-JNK). After incubation with secondary antibodies, protein expression was visualized using enhanced chemiluminescence reagents and a Tanon-5200 Multi Gel Imaging System (Tanon Science and Technology). The intensities of the protein bands were quantified with the Gel-Pro Analyzer 4.0 software (Media Cybernetics) and normalized to that of β-actin (ABclonal Technology Co., Ltd., Wuhan, China; AC038, 1:50,000 dilution in 5% bovine serum albumin) as an internal control to ensure the accuracy of the results.

### Statistical analysis

All of the data are presented as mean ± SD. The GraphPad Prism 7.0 software (GraphPad, San Diego, CA) was used for the statistical analysis. Differences between 2 samples were analyzed with the Student *t* test, whereas differences among multiple groups were compared using one-way analysis of variance; Tukey correction was used to conduct post hoc multiple comparisons of the specific differences among each group. *P* < 0.05 or *P* < 0.01 was considered statistically significant.

## Results

### Tasq is a potential drug for the treatment of AP, but it comes with certain toxicity

**Fig. 2.**
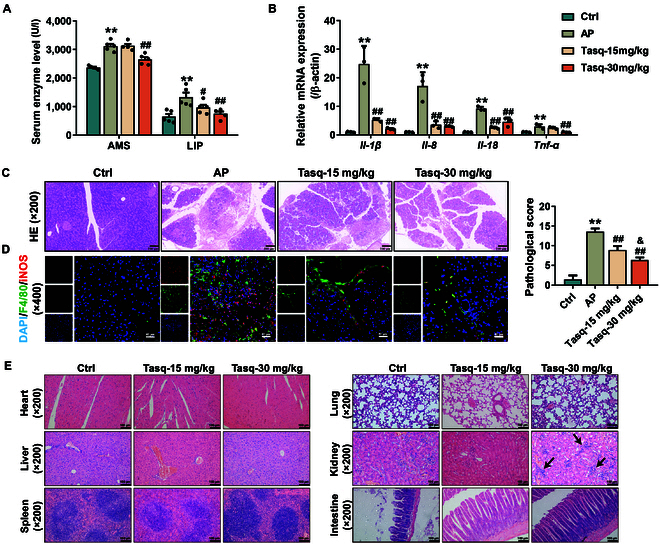
Tasquinimod (Tasq) relieves acute pancreatitis (AP)-induced pancreatic damage and inflammatory response, but doses of Tasq-30 mg/kg cause drug toxicity in mice. (A) The serum levels of amylase (AMS) and lipase (LIP) in mice (*n* = 5). (B) The relative messenger RNA (mRNA) expression of inflammatory factors in the pancreatic tissue (*n* = 3). (C) Hematoxylin and eosin (HE) staining and pathology score of pancreas (*n* = 5). (D) Immunofluorescence detecting the content of M1-like macrophages in pancreatic tissue. (E) HE staining of mouse heart and liver, spleen, lung, kidney, and intestine. Data are expressed as mean ± SD; **P < 0.01 vs. control (Ctrl), ^#^*P* < 0.05 and ^##^*P* < 0.01 vs. AP, and ^&^*P* < 0.05 vs. Tasq-15 mg/kg. DAPI, 4′,6-diamidino-2-phenylindole.

An AP mouse model was established by retrograde injection of STC into the biliopancreatic duct to investigate the therapeutic effect of Tasq on AP. To our knowledge, no similar studies have been reported to date. As shown in Fig. 2A, the serum levels of AMS and LIP were greater in AP mice than in control (Ctrl) mice. Compared with AP, Tasq-15 mg/kg significantly reduced only serum LIP levels without affecting serum AMS levels, but Tasq-30 mg/kg sensibly decreased both serum LIP and AMS levels. Moreover, the mRNA expression levels of the inflammatory factors, *Il-1β*, *Il-8*, and *Il-18*, in the pancreas of AP mice were significantly greater than those in the Ctrl mice but were significantly lower after Tasq treatment. Interestingly, *Tnf-α* mRNA expression was obviously up-regulated by AP, but was significantly down-regulated after treatment with Tasq-30 mg/kg (Fig. 2B). Pathology and immunofluorescence of the pancreas revealed that Tasq ameliorated the tissue damage and inflammatory infiltration induced by AP by decreasing the number of M1-like macrophages in a dose-dependent manner and reducing the pathological score of the pancreas (Fig. 2C and D). The above results demonstrate that Tasq can alleviate the pancreatic pathological damage and inflammatory response induced by AP, and it shows better efficacy at a dose of 30 mg/kg.

To evaluate the in vivo toxicity of Tasq, the serum BUN and CRE (renal toxicity indicators) and ALT and AST (liver toxicity indicators) in mice were measured, and histopathological examination of heart, liver, spleen, lung, kidney, and intestinal tissues was conducted as well. HE staining confirmed that there was no notable toxicity of Tasq at various doses on the heart, liver, spleen, lungs, and intestines of healthy mice. Similarly, the levels of BUN, CRE, ALT, and AST in the mouse serum show no significant increase compared to those of the Ctrl group (Fig. S1). It is worth noting that at a dose of 30 mg/kg Tasq, inflammatory cell infiltration in the renal tissue could be observed (Fig. 2E). These results suggest that doses of 30 mg/kg may exert toxic effects on the kidneys, consistent with the nephrotoxicity found in Tasq’s phase II clinical trials. Therefore, the toxicity and efficacy experiments demonstrated that Tasq exhibits better therapeutic effects at a dose of 30 mg/kg, but it also has toxic side effects.

### Polypeptides targeting M1-like macrophages

**Fig. 3.**
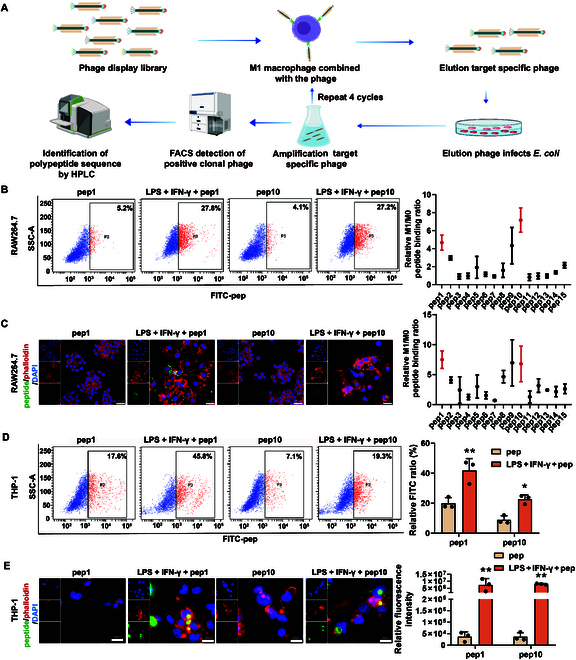
Peptides targeted M1-like macrophages (M1peps). (A) Schematic diagram of phage display library screening for positive targeting peptides. (B) Flow cytometry for detecting the targeting of peptide sequences to M1-polarized RAW264.7 cells (*n* = 3). (C) Laser confocal imaging for detecting the targeting of peptide sequences to M1-polarized RAW264.7 cells (*n* = 3). (D) Flow cytometry for detecting the targeting of peptide sequences to M1-polarized THP-1 cells (*n* = 3). (E) Laser confocal imaging for detecting the targeting of peptide sequences to M1-polarized THP-1 cells (*n* = 3). Data are expressed as mean ± SD; *P < 0.05 and **P < 0.01 vs. pep. HPLC, high-performance liquid chromatography; FACS, fluorescence-activated cell sorting; *E. coli*, *Escherichia coli*; LPS, lipopolysaccharide; IFN-γ, interferon-γ; FITC, fluorescein isothiocyanate.

**Fig. 4.**
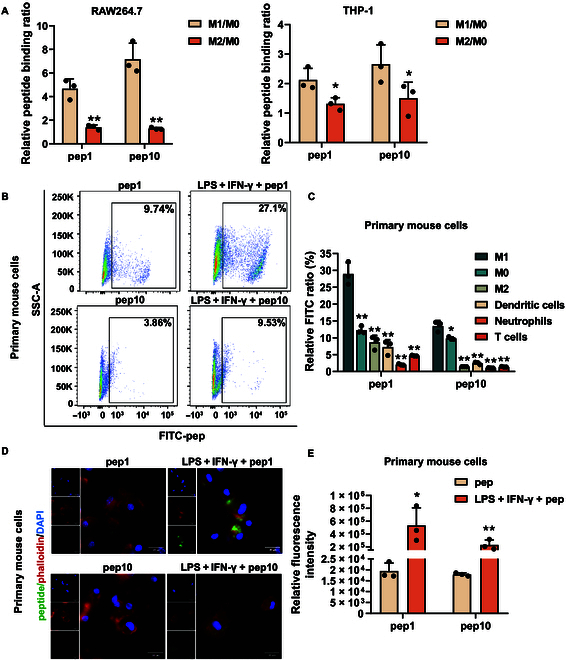
Peptides targeted M1peps. (A) Flow cytometry was used to detect the ratio of M1-type polarization to M2-type polarization in RAW264.7 cells and THP-1 cell targeted by peptide sequences (*n* = 3). (B and C) Flow cytometry was used to detect the targeting effects of peptide sequences on M1-type polarized bone-marrow-derived macrophages (BMDMs), unpolarized BMDMs, M2-type-polarized BMDMs, mouse bone-marrow-derived dendritic cells (DCs), mouse peripheral blood T cells, and neutrophils (*n* = 3). (D and E) Laser confocal imaging for detecting the targeting of peptide sequences to M1-polarized BMDMs (*n* = 3). Data are expressed as mean ± SD; *P < 0.05 and **P < 0.01 vs. pep or M1.

Peptides that target M1-like macrophages were identified by phage display technology, and the screening diagram is shown in Fig. 3A and Figs. S2 and S3. A total of 6 heptapeptides and 9 dodecapeptides were obtained as positive sequences (Table S2). FITC-pep complexes were constructed by conjugating 15 synthesized peptide sequences with FITC fluorescent groups and were added to RAW264.7 cells with or without LPS + IFN-γ stimulation (inducers of M1 macrophage polarization). Flow cytometry and laser confocal microscopy revealed that the binding ratios of M1-like macrophages in the M1pep1 and M1pep10 sequences were greater than those in the other sequences (Fig. 3B and C). Subsequently, human macrophages were used to further validate the targeting of M1pep1 and M1pep10. After inducing THP-1 cells to become M1-like macrophages (Fig. S4A), flow cytometry and laser confocal microscopy revealed that both M1pep1 and M1pep10 also exhibited targeting specificity for M1-type THP-1 cells (Fig. 3D and E). Moreover, M1pep1 and M1pep10 exhibited remarkably stronger targeting effects on M1-type RAW264.7 and THP-1 cells than on M2-type RAW264.7 and THP-1 cells (Fig. 4A and Fig. S4B to E). To further verify the targeting specificity of M1pep, we extracted immune cells from the peripheral blood of mice as well as bone marrow cells from mice and induced the mouse bone marrow cells into macrophages and DCs. T cells were characterized using CD3, neutrophils were characterized using CD11b and Ly6G, and DCs were characterized using CD11c and MHC-II. As shown in Fig. 4B to E and Fig. S5A to F, M1pep1 and M1pep10 exhibited targeting specificity toward M1-type primary mouse macrophages.

### Design and synthesis of M1pep-Tasq

First, the structure of Tasq was modified to improve its ability to bind to targeted peptides through linkers. The restructuring process and ^1^H nuclear magnetic resonance (HNMR) and high-performance liquid chromatography (HPLC) data of the reconstructed Tasq are shown in the Supplementary Methods and Figs. S6 to S8, and the structure of the modified Tasq is shown in Fig. 5A. Second, the modified Tasq was conjugated with the polypeptide M1pep1 or M1pep10 through the flexible connector GGGSKKK. The coupling process is described in Fig. 5B. The HNMR and HPLC data of NCpep-Tasq, M1pep1-Tasq, and M1pep10-Tasq are shown in Figs. S9 to S14.

**Fig. 5.**
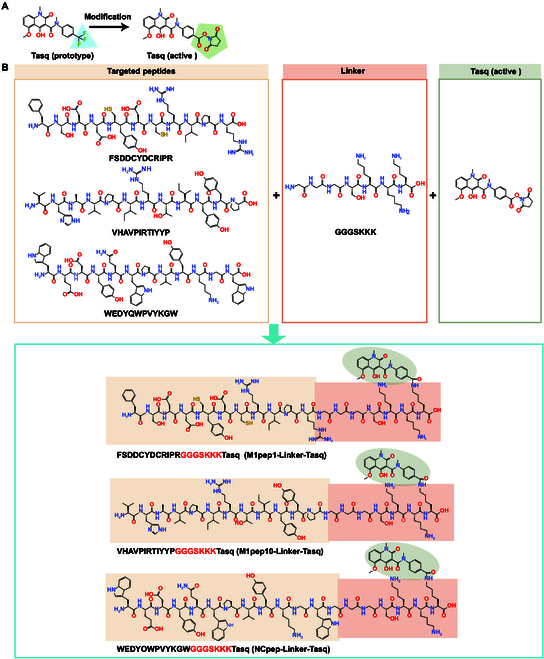
Design and synthesis of M1pep-Tasq. (A) Structural modification of Tasq. (B) Synthesis of M1pep1-Tasq, M1pep10-Tasq, and NCpep-Tasq.

### M1pep-Tasq reduces toxicity and enhances efficacy

The above experiments showed that Tasq has therapeutic effects on AP, but it comes with toxic side effects. Therefore, we developed a new type of PDC drug—M1pep-Tasq—with the aim of improving the efficacy of Tasq and reducing its toxicity. To compare the in vivo drug toxicity of M1pep-Tasq and Tasq, the tissue structure of the heart, liver, spleen, lungs, kidneys, and intestine in mice was examined, along with liver toxicity indicators (ALT and AST), cardiac and skeletal muscle toxicity indicators (creatine kinase), and renal toxicity indicators (CRE, BUN, and uric acid [UA]) in mouse serum. The results showed that Tasq exhibited notable nephrotoxicity. HE staining revealed that Tasq-30 and NCpep-Tasq-30 caused glomerular shrinkage, damage to the renal tubular structure, and immune cell infiltration (Fig. 6A). Compared to the AP group mice (64.03 ± 15.83 mM), Tasq-30 (135.30 ± 16.03 mM), and NCpep-Tasq-30 (130.80 ± 21.07 mM) significantly increased BUN levels in mouse serum, while UA showed a downward trend (AP group: 52.55 ± 8.30 mg/l; Tasq-30: 40.51 ± 6.24 mg/l) (Fig. 6B and C). There were no marked differences in other toxicity indicators. However, M1pep-Tasq showed no notable toxicity (Fig. S15A to C). The above results indicate that M1pep-Tasq can reduce the in vivo toxicity of Tasq.

**Fig. 5.**
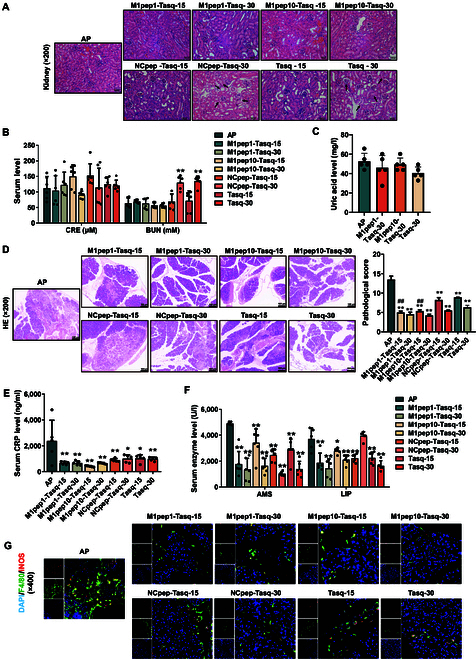
M1pep-Tasq reduces the toxicity of Tasq and enhances its efficacy by improving its targeting ability. (A) HE staining of the mouse kidney. (B) The serum levels of kidney function markers, creatinine (CRE) and blood urea nitrogen (BUN) (*n* = 6). (C) The serum levels of kidney function markers, uric acid (UA) (*n* = 5). (D and E) HE staining of the pancreas (*n* = 6). (E) The serum levels of acute inflammatory markers, C-reactive protein (CRP) (*n* = 5). (F) The serum levels of AMS and LPS in mice (*n* = 6).(G) Immunofluorescence detecting the content of M1peps in pancreatic tissue. Data are expressed as mean ± SD; *P < 0.05 and **P < 0.01 vs. AP, and ^##^*P* < 0.01 vs. Tasq-15.

A comparison of the effects of Tasq and M1pep-Tasq on improving pancreatic pathological injury in AP mice revealed that M1pep-Tasq had a better effect on improving AP-induced pancreatic edema, hemorrhage, acinar cell necrosis, and inflammatory cell infiltration than did Tasq alone or NCpep-Tasq at the same dose (Fig. 6D). As shown in Fig. 6E and F, compared with those in AP mice, the levels of serum CRP, AMS, and LIP were all significantly lower after treatment with Tasq, M1pep-Tasq, and NCpep-Tasq. Immunofluorescence showed that compared with Tasq or NCpep-Tasq alone, M1pep-Tasq was more effective at reducing the polarization of macrophages to the M1 type in the pancreas of AP mice (Fig. 6G).

### M1pep-Tasq specifically targets pancreatic cells to inhibit the M1 polarization of macrophages

**Fig. 7.**
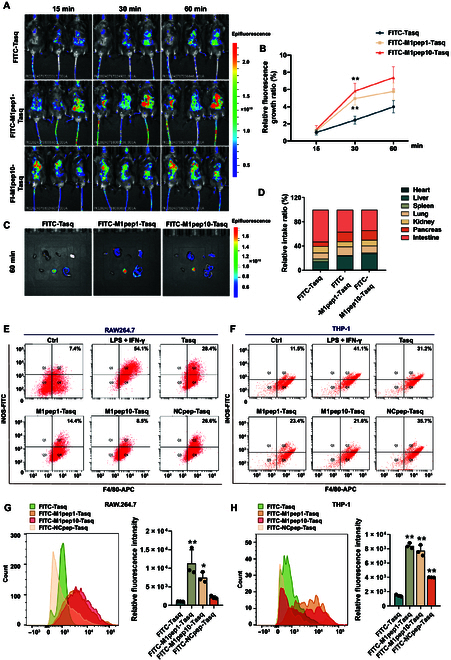
M1pep-Tasq enhances the efficacy of Tasq by enhancing the targeting ability of Tasq. (A and B) Distribution of FITC-Tasq, FITC-M1pep1-Tasq, and FITC-M1pep10-Tasq in AP mice after 15, 30, and 60 min of administration. (C and D) Distribution of FITC-Tasq, FITC-M1pep1-Tasq, and FITC-M1pep10-Tasq in different tissues in AP mice after 60 min of administration. (E and F) Flow cytometry for detecting the ratio of M1-like macrophages (*n* = 3). (G and H) Flow cytometry detection of M1-like macrophage targeting of FITC-Tasq, FITC-M1pep1-Tasq, FITC-M1pep10-Tasq, and FITC-NCpep-Tasq (*n* = 3). Data are expressed as mean ± SD; *P < 0.05 and **P < 0.01 vs. FITC-Tasq.

To verify the tissue-targeting ability of M1pep-Tasq in vivo, the fluorescent label FITC was attached to Tasq, M1pep1-Tasq, and M1pep10-Tasq. Next, equal amounts of FITC-Tasq, FITC-M1pep1-Tasq, and FITC-M1pep10-Tasq were injected into AP mice via the tail vein. The HNMR and HPLC data of FITC-Tasq, FITC-M1pep1-Tasq, and FITC-M1pep10-Tasq are shown in Figs. S16 to S21. In vivo imaging revealed that the fluorescence intensity and relative fluorescence growth ratio of FITC-M1pep1-Tasq and FITC-M1pep10-Tasq within 1 h after administration were significantly greater than those of FITC-Tasq. These findings indicate that M1pep-Tasq has better tissue penetration ability than unmodified Tasq (Fig. 7A and B). The fluorescence in various organs was subsequently observed 1 h after administration, and the proportion of FITC-M1pep1-Tasq and FITC-M1pep10-Tasq fluorescence in the pancreas was significantly greater than that of FITC-Tasq, whereas the proportion of fluorescence in the kidneys was significantly lower. These findings indicate that M1pep-Tasq enhances the targeting of Tasq to pancreatic tissue in vivo and reduces nephrotoxicity (Fig. 7C and D).

During in vitro experiments, the safe dose of Tasq, M1pep1-Tasq, M1pep10-Tasq, and NCpep-Tasq for mouse RAW264.7 macrophages was determined. The analysis of cell viability revealed that when the drug concentration did not exceed 20 μM, none of the 4 conjugates exhibited notable cytotoxicity to mouse macrophages (Fig. S22A). As shown in Fig. 7E and F and Fig. S22B and C, Tasq administration significantly reduced the proportion of M1 polarization in RAW264.7 cells and THP-1 cells induced by LPS + IFN-γ stimulation and inhibited the gene expression of the M1-like macrophage markers *iNos*, *Cd86*, and *Tnf-α*. As expected, compared with Tasq, M1pep-Tasq had a greater inhibitory effect on the polarization of M1-like macrophages, but the inhibitory effect of NCpep-Tasq was roughly equivalent to that of Tasq. The results of immunofluorescence and flow cytometry revealed that the fluorescence density in mouse and human macrophages treated with M1pep-Tasq was significantly greater than that in macrophages treated with Tasq or NCpep-Tasq (Fig. 7G and H and Fig. S22D and E). These results suggest that M1pep-Tasq can increase the ability of Tasq to target M1-like macrophages, improving its efficacy in inhibiting the polarization of M1-like macrophages.

### M1pep-Tasq inhibits macrophage M1 polarization by inhibiting the S100A9–TLR4–MAPK pathway

**Fig. 8.**
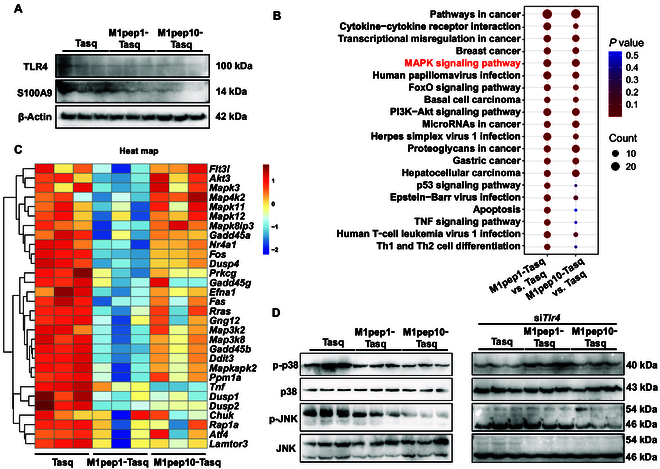
M1pep-Tasq inhibits macrophage M1 polarization by inhibiting the S100A9–TLR4–MAPK pathway. (A) Protein expression of S100A9 and TLR4 in RAW264.7 cells. (B) KEGG enrichment analysis of DEGs. (C) Heat map of DEGs related to the MAPK signaling pathway. (D) Expression of p38 MAPK, p-P38 MAPK, JNK, and p-JNK in RAW264.7 cells.

As Tasq is a specific inhibitor of S100A9, the regulatory effects of different doses of M1pep-Tasq on the protein expression of S100A9 and its ligand TLR4 in macrophages were detected by Western blotting. Compared with Tasq, M1pep-Tasq effectively reduced the expressions of S100A9 and TLR4 proteins (Fig. 8A and Fig. S23A). To further explore the mechanism by which M1pep-Tasq enhances the inhibitory effect of Tasq on M1 macrophage polarization, RNA sequencing analysis was used to compare differential gene expression in RAW264.7 cells treated with M1pep1-Tasq, M1pep10-Tasq, or Tasq. As shown in Fig. S23B and C, compared with Tasq, M1pep1-Tasq induced the up-regulation of 347 genes and the down-regulation of 545 genes, whereas M1pep10-Tasq induced the up-regulation of 395 genes and the down-regulation of 157 genes. There were 138 DEGs between the M1pep1-Tasq and M1pep10-Tasq groups. Kyoto Encyclopedia of Genes and Genomes analysis of the DEGs suggested that the MAPK signaling pathway may be a key downstream pathway for the enhanced pharmacological action of M1pep-Tasq in inhibiting M1 macrophage polarization (Fig. 8B). Heat maps of DEGs and quantitative polymerase chain reaction (qPCR) results revealed that the expression levels of genes related to the MAPK signaling pathway, such as *flt3l*, *akt3*, *mapk3*, *map4k2*, *mapk11*, *mapk12*, and *mapk8ip3*, were significantly lower after M1pep-Tasq administration than after Tasq alone (Fig. 8C and Fig. S23D). To further verify the impact of M1pep-Tasq on the MAPK signaling pathway, we used Western blotting to detect the expression levels of key proteins in the MAPK signaling pathway, including p38 MAPK and JNK in RAW264.7 cells. Compared with Tasq, M1pep-Tasq effectively reduced the phosphorylation of the p38 and JNK proteins (Fig. 8D and Fig. S23E). Numerous studies have already demonstrated that the MAPK signaling pathway can be activated by TLR4 [15–17]. To further confirm that the MAPK pathway is downstream of TLR4, we used a si*Tlr4* interference plasmid to reduce the expression of TLR4 in RAW264.7 cells. We verified the knockdown efficiency of siTlr4 by PCR and selected the sequence with the highest knockdown efficiency for subsequent validation (Fig. S23F). As shown in Fig. 8D and Fig. S22G, we found that compared with those in the si*Tlr4* + Tasq group, there was no marked difference in the phosphorylation levels of the p38 and JNK proteins in the si*Tlr4* + M1pep-Tasq group, suggesting that the MAPK pathway is downstream of TLR4. These results indicate that M1pep-Tasq may exert its effects by targeting the S100A9–TLR4–MAPK pathway.

## Discussion

Current interventions alleviate only symptoms, but medications for treating AP are far from achieving satisfactory endpoints [18,19]. The development of new drugs typically takes 10 to 15 years. Despite rigorous selection in the preclinical phase, up to 90% of drug candidates fail during clinical trials [20,21]. Comfortingly, drug repurposing increases drug utilization by discovering new uses for existing drugs, minimizing the costs, time, and risks of new drug development to accelerate the transition from basic research to clinical application and bring more therapeutic benefits to patients [22].

Tasq is a drug designed initially to target hormone-resistant advanced prostate cancer and may have notable potential as a new clinical drug for the treatment of AP due to its specific inhibition of S100A9 [23]. As expected, in vivo pharmacological experiments revealed for the first time that Tasq significantly improved AP-induced pancreatic injury in a dose-dependent manner and reduced the serum AMS, LIP, and inflammatory factor levels. This work increases our confidence that developing Tasq as a candidate drug for treating AP is a worthwhile endeavor. The frustrating reality is that Tasq did not pass phase III clinical trials, with long-term use of Tasq leading to a range of severe adverse drug reactions, such as kidney and urinary tract disorders (7.3%), infections and invasions (5.1%), and hematologic and lymphatic disorders (4.3%) [8,9]. Moreover, our research revealed that although 30 mg/kg Tasq is more effective at treating AP than 15 mg/kg Tasq is, it is also accompanied by certain types of renal toxicity.

To reduce systemic toxicity and increase the therapeutic efficacy of Tasq, we used drug-targeting strategies. PDCs offer a novel approach to achieving precise drug delivery to target cells, maximizing therapeutic effects while minimizing systemic toxicity [12,24]. Since the 2018 US FDA approval of Lutathera (developed by Novartis)—the world’s first PDC drug—for treating somatostatin-receptor-positive gastroenteropancreatic neuroendocrine tumors [25], the field has experienced accelerated development. Multiple novel PDC agents are currently in clinical development, including TH1902 (targeting SORT1-positive advanced solid tumors, phase I trial), ANG1005 (for breast cancer brain metastases, phase II trial), and BT8009 (targeting nectin-4, phase I/II clinical trials) [26–28]. These advancements provide strong momentum for PDC technology translation. PDCs comprise 3 core components: a targeting peptide, a cleavable linker, and a therapeutic payload. Their mechanism relies on enzyme-responsive linkers designed to ensure selective payload release within target cells. As macrophages are the primary source of the pro-inflammatory factor S100A9 and M1-polarized macrophages drive the inflammatory cascade in AP through the release of pro-inflammatory mediators [4,19], this study focused on developing a PDC delivery system that targets M1 macrophages. By directing Tasq specifically to M1 macrophages within the pathological microenvironment, we aimed to reduce systemic exposure toxicity while increasing its efficacy in alleviating AP.

PDT is an efficient screening method in drug development that can select specific peptides as targeted ligands for precise drug delivery, increasing drug efficacy and reducing side effects. By phage display technology and experimental verification, we ultimately determined the 2 most specific peptide sequences, M1pep1 (FSDDCYDCRIPRP) and M1pep10 (VHAVPIRTIYY). M1pep1 and M1pep10 were subsequently conjugated with activated Tasq using a flexible linker (GGGSKKK) to form novel PDCs, M1pep-Tasq.

To assess the toxicity and efficacy of the new drug, we administered M1pep-Tasq to AP model mice via intraperitoneal injection. HE staining and serological indicators revealed that both low and high doses of Tasq have nephrotoxicity, but M1pep-Tasq (Tasq doses of 15 and 30 mg/kg) can alleviate these side effects. Compared with Tasq alone or the same dose of NCpep-Tasq, M1pep resulted in greater improvement in AP-related damage, especially at a dose of 30 mg/kg. Both M1pep1-Tasq and M1pep10-Tasq can significantly improve AP, reducing the dosage of Tasq administered by significantly increasing the therapeutic effect of Tasq. We subsequently evaluated the in vivo targeting ability of M1pep-Tasq. In vivo imaging revealed that at the same time point after administration, M1pep-Tasq had better tissue penetration than unmodified Tasq did, and 1 h after administration, M1pep-Tasq enhanced the pancreatic targeting of Tasq. We also observed that the targeting of M1pep-Tasq to the liver was increased, possibly due to the liver being the main site of drug metabolism. Considering that all doses of Tasq have no effect on liver function or liver tissue, they are nontoxic to the liver.

Furthermore, in vitro results from qPCR, immunofluorescence, and flow cytometry assays revealed that under the same conditions, M1pep-Tasq enhanced the ability of Tasq to target M1-like macrophages and better suppressed the polarization of macrophages toward the M1 phenotype within the cells. These findings indicate that M1pep-Tasq can effectively deliver drugs to M1-type macrophages, regardless of whether the macrophages are of mouse or human origin.

We also explored the mechanism by which M1pep-Tasq reduces toxicity and increases efficacy. RNA sequencing followed by Western blot validation indicated that M1pep-Tasq may regulate the MAPK/JNK pathway by inhibiting the expressions of S100A9 and TLR4. S100A9 is considered a ligand for TLR4, which stimulates a pro-inflammatory response by activating the MAPK pathway. The p38 MAPK signaling pathway plays a key role in regulating inflammatory responses [29]. In its inactive state, MAPK is primarily localized in the cytoplasm, and once activated, it translocates to the nucleus, where it phosphorylates various transcription factors, thereby regulating the expression of multiple effector molecules, including cytokines [30,31]. In the context of AP, the activation of p38 MAPK increases the production of inflammatory mediators such as TNF-α and IL-1β, triggering a cascade of reactions that lead to an imbalanced inflammatory response [32,33]. Studies have also shown that inhibiting the MAPK pathway can effectively reduce the release of inflammatory mediators and inhibit M1 polarization of macrophages, thereby alleviating the symptoms of AP [34].

In this study, we leveraged the advantages of PDT to identify M1pep, which can specifically target M1-type macrophages. We successfully synthesized a novel PDC drug, M1pep-Tasq, by coupling M1pep with activated Tasq via a flexible linker. M1pep-Tasq increases the targeting of Tasq to M1-like macrophages and damaged pancreatic tissue, significantly improving the efficacy of Tasq in treating AP and reducing its drug toxicity. The mechanism of the reduced toxicity and increased efficacy of M1pep-Tasq may involve the inhibition of M1-like macrophage polarization by the suppression of the S100A9–TLR4–MAPK pathway.

## Data Availability

All data generated during this study leading to the findings presented here are included in this published article and its supplementary data files. All data are available from the corresponding author upon reasonable request.
