# Supplementary material for: The Peptide–Drug Conjugate M1pep–Tasquinimod Ameliorates Acute Pancreatitis via Selectively Clearing M1-like Macrophages
Source: Biomater Res. 2025 Sep 24;29:0250. doi: 10.34133/bmr.0250 (PMC12457742; doi:10.34133/bmr.0250)
Supplement: Supplementary 1 — Supplementary Methods Figs. S1 to S23 Tables S1 and S2 [file bmr.0250.f1.pdf]

Supplementary Materials for

The Peptide-Drug Conjugate M1pep-Tasquinimod Ameliorates Acute Pancreatitis Via Selectively Clearing M1-like Macrophages

Fangyue Guo *et al.*

Corresponding author: Hong Xiang, xianghong@dmu.edu.cn

This PDF file includes:

Methods
Figures. S1 to S23
Tables S1 to S2

1. Methods

1.1. Peptide synthesis

In the process of solid phase peptide synthesis (SPPS), AM resin was first placed into a reaction column and activated by sonication with dichloromethane (DCM). Then, protected amino acids, O-Benzotriazol-1-yloxytris (pyrrolidino) phosphonium hexafluorophosphate (HBTU), and N,N-Diisopropylethylamine (DIEA) were sequentially added to carry out the coupling reaction. The coupling was checked with a piperidine/dimethylformamide (DMF) solution to ensure the reaction was successful. This process was repeated until all amino acids were linked. Afterwards, the resin was washed with DMF, DCM, and methanol. Finally, the resin was treated with a cleavage solution prepared with trifluoroacetic acid (TFA) and other reagents to release the peptide, which was then purified through precipitation and centrifugation with diethyl ether, followed by multiple washes to obtain the pure peptide product.

1.2. Structural modification of Tasq

Structural modification of Tasq was shown in the **Figure S6**. The main steps are as follows:

Step 1: In a single-neck flask, slowly add 38% concentrated hydrochloric acid (HCl, 3 mL) to glacial acetic acid (9 mL), then add compound 1 (1 g, 1 eq), and heat to 70 °C for 18 hours. After the reaction, cool to room temperature, filter, and wash with ethanol (EtOH, 20 mL). Dry to obtain white solid compound 2 (0.6 g, yield 66%).

Step 2: In a single-neck flask, sequentially add HATU (1-[Bis(dimethylamino)methylene]-1H-1,2,3-triazolo[4,5-b]pyridinium 3-oxid hexafluorophosphate, 1.83 g, 2 eq) and DIPEA (N,N-Diisopropylethylamine, 0.94 g, 3 eq) to a DMF (Dimethylformamide, 10 mL)/THF (Tetrahydrofuran, 10 mL) solution containing compound 2 (0.6 g, 1 eq). Stir at room temperature for 10 minutes, then add compound 2a (0.48 g, 1.2 eq) and stir at 50 °C for 2 hours. After the reaction, adjust pH to 6-7, concentrate, and purify using a Kromasil C18-5 HPLC column (20%-55% MeCN, 0.1% TFA). Lyophilize to obtain yellow oily compound 3 (0.5 g, yield 52.6%).

Step 3: In a single-neck flask, add a 5 mL aqueous solution of KOH (Potassium hydroxide, 0.21 g, 3 eq) to a THF (10 mL) solution containing compound 3 (0.5 g, 1 eq), and stir at 50° C for 4 hours. After the reaction, adjust pH to 5-6, concentrate, and pour off the water. Add anhydrous methanol (MeOH) to the yellow viscous

residue at the bottom, sonicate, filter, and dry to obtain white solid compound 4 (0.2 g, yield 41.5%).

Step 4: Add DCC (Dicyclohexylcarbodiimide, 0.13 g, 1.2 eq) to a DMF (5 mL) suspension of compound 4 (0.2 g, 1 eq), followed by compound 4a (90 mg, 1.5 eq) and DMAP (4-Dimethylaminopyridine, 6.4 mg, 0.1 eq), and stir at 50 °Cfor 2 hours. Filter and purify using a Kromasil C18-5 HPLC column (20%-50% MeCN, 0.1% TFA). Lyophilize to obtain white solid modified tasquinimod (0.12 g, yield 48%, purity 97.8%, M+H+=480.1).

Nuclear Magnetic Resonance Hydrogen spectroscopy (HNMR) and High-Performance Liquid Chromatography (HPLC) were utilized to confirm the target product. The HNMR data for the target product after structural modification is presented in **Figure S7**. 1H NMR (400 MHz, CDCl3) δ 9.91 (s, 1H), 8.00 (d, J = 8.6 Hz, 2H), 7.50 (dd, J = 17.7, 8.6 Hz, 3H), 6.98 (d, J = 8.7 Hz, 1H), 6.72 (d, J = 8.2 Hz, 1H), 4.06 (s, 3H), 3.59 (s, 3H), 3.53 (s, 3H), 2.91 (d, J = 4.4 Hz, 4H). The HPLC data for the target product after structural modification is shown in **Figure S8**.

1.3. Chemical Synthesis of FITC-M1pep-Tasq

Fmoc-ACP Coupling: Add a threefold molar amount of Fmoc-ACP and HBTU to the resin, dissolve with a small amount of DMF, then immediately add a tenfold molar amount of DIEA, and react for 30 minutes. After the reaction, remove the solvent, take a few resin beads, wash with ethanol three times, then add one drop each of ninhydrin, pyridine, and phenol, and heat to 105 °C-110 °C for 5 minutes. If the resin remains colorless, the reaction is successful; if it turns blue, re-coupling is necessary. After confirming successful coupling, wash the resin twice with 15 mL each of DMF and methanol.

Deprotection: First, add 20 mL of a 20% piperidine/DMF solution, remove it after 5 minutes. Then add another 20 mL of the same solution and shake for 15 minutes. After removing the piperidine solution, wash the resin beads with ethanol three times, add one drop each of ninhydrin, pyridine, and phenol, and heat to 105 °C-110 °C for 5 minutes. If the resin turns deep blue, deprotection is complete, and you can proceed to couple FITC; if there is no color change, deprotection is incomplete, and you need to repeat the process. After confirming successful deprotection, wash the resin twice with 15 mL each of DMF and methanol.

FITC Coupling: Add a threefold molar amount of FITC to the resin, dissolve

with a small amount of DMF, then immediately add a tenfold molar amount of DIEA, and react for 60 minutes. After the reaction, remove the solvent, wash the resin beads with ethanol three times, add one drop each of ninhydrin, pyridine, and phenol, and heat to 105 °C-110 °C for 5 minutes. If the resin remains colorless, FITC coupling is successful; if it turns blue, re-coupling is necessary. After confirming successful FITC coupling, wash the resin twice with 15 mL each of DMF and methanol.

Peptide Folding: After the FITC coupling is complete, the folding reaction is finished. Proceed to the final washing stage, wash with DMF three times, DCM three times, and methanol three times, then remove the solvent from the peptide resin.

Side Chain Deprotection and Resin Cleavage: Prepare 15 mL of cleavage solution with the volume ratio of TFA (94.5%), water (2%), EDT (2.5%), and TIS (1%). Load the resin into a flask and oscillate at 30 °C for 2 hours. Blow dry the cleavage solution with nitrogen as much as possible, then pour it into a centrifuge tube and slowly add ether. Cap and centrifuge for 5 minutes, discard the supernatant, and the white solid remains at the bottom. Wash with ether six more times, then evaporate at room temperature to obtain the crude peptide product.

1.4. Biochemical index detection

Amylase (AMS) detection

The substrate buffer was pre-warmed to 37 °C for a duration of 5 min. Serum samples from each group of mice were divided into two subsets: one for measurement and one for blank. The measurement subset's serum was combined with the substrate buffer, whereas the blank subset received only the substrate buffer. After being incubated at 37 °C for 7.5 min, 200 μL of a pre-mixed solution of iodine and deionized water was added to each well of both the measurement and blank groups. Subsequently, the absorbance at 660 nm was measured for both sets using a microplate reader (BioTek Cytation3, US).

Lipase (LIP) detection

The content of LIP in mouse serum was detected according to the instructions of the LPS assay kit (Njjcbio; Nanjing, China). The serum was added to the substrate buffer, which had been pre-warmed at 37 °C for 5 min and was quickly mixed. Then, 200 μL of this mixture was added to each well of a 96-well plate, and the absorbance

at 420 nm was measured using a microplate reader (BioTek Cytation3, US).

Creatinine (CRE) detection

The content of CRE in mouse serum was detected according to the instructions of the CRE assay kit (Njjcbio; Nanjing, China). For the detection of serum CRE, blank wells, standard wells, and measurement wells were prepared, with 6 μL of deionized water, standard solution, or serum to be tested being added as appropriate. Subsequently, 180 μL of Solution A was added to these wells. After incubation at 37 °C for 5 min, the absorbance was measured at a wavelength of 546 nm. Then, 60 μL of Enzyme Solution B was added to the wells, mixed thoroughly, and the reaction was allowed to proceed at 37 °C for an additional 5 min, after which the absorbance was read again.

Blood urea nitrogen (BUN) detection

The content of BUN in mouse serum was detected according to the instructions of the BUN assay kit (Njjcbio; Nanjing, China). The assay was divided into blank, standard, and measurement groups, to which 200 μL of deionized water, standard solution, or serum to be tested were added, respectively. Then, 250 μL of buffer enzyme solution was added, and the mixture was incubated at 37 °C for 10 min. Subsequently, 1 mL of phenol chromogenic reagent and 1 mL of alkaline sodium hypochlorite were added, and the reaction was carried out at 37 °C for 10 min. The absorbance was read at a wavelength of 640 nm.

Alanine aminotransferase (ALT) and Aspartate aminotransferase (AST) detection

The content of ALT or AST in mouse serum was detected according to the instructions of the ALT or AST assay kit (Njjcbio; Nanjing, China). Initially, an ALT or AST standard curve was established. To each tube, 500 μL of substrate solution was added, and to the sample wells, 100 μL of serum was introduced. The reaction proceeded at 37 °C for 30 min. Following this, 500 μL of 2,4-dinitrophenylhydrazine solution was incorporated, and the mixture was incubated at 37 °C for an additional 20 min. Then, 5 mL of sodium hydroxide solution was added. The absorbance was measured at a wavelength of 505 nm using a microplate reader (BioTek Cytation3,

US). The ALT content in the serum to be tested was calculated using the formula derived from the standard curve.

Uric acid (UA) detection

The content of UA in mouse serum was detected according to the instructions of the UA assay kit (Njjcbio; Nanjing, China). To each tube, 200 µL of serum was added, followed by the addition of 2 mL of tungstic acid protein precipitant. The mixture was thoroughly mixed and then allowed to stand for 10 min before being centrifuged. Subsequently, 1.6 mL of the supernatant was collected, and 0.5 mL of CUT reagent along with 0.5 mL of phosphotungstic acid reagent were introduced. The mixture was then left to stand at room temperature for 10 min, after which the absorbance was measured at 690 nm.

Creatine kinase (CK) detection

According to the instructions of the CK assay kit (Njjcbio; Nanjing, China), the content of CK in mouse serum was determined. Serum from each group of mice was divided into a measurement group and a control group. The serum in the measurement group was combined with the substrate buffer, whereas only the substrate buffer was added to the control group. The mixtures were then incubated at 37 °C for 20 min before being centrifuged to separate the supernatant. The supernatant from both the measurement and control groups was subsequently mixed with the pre-mixed phosphorus reagent. Finally, the absorbance was measured at 660 nm using a microplate reader (BioTek Cytation3, US).

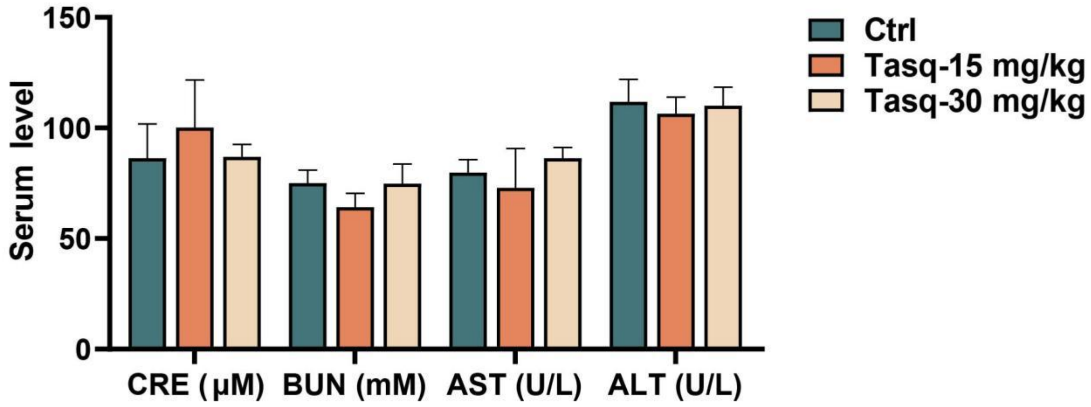

Figure S1 The serum levels of kidney function markers (CRE and BUN), liver

function markers, (ALT and AST) (n=6).

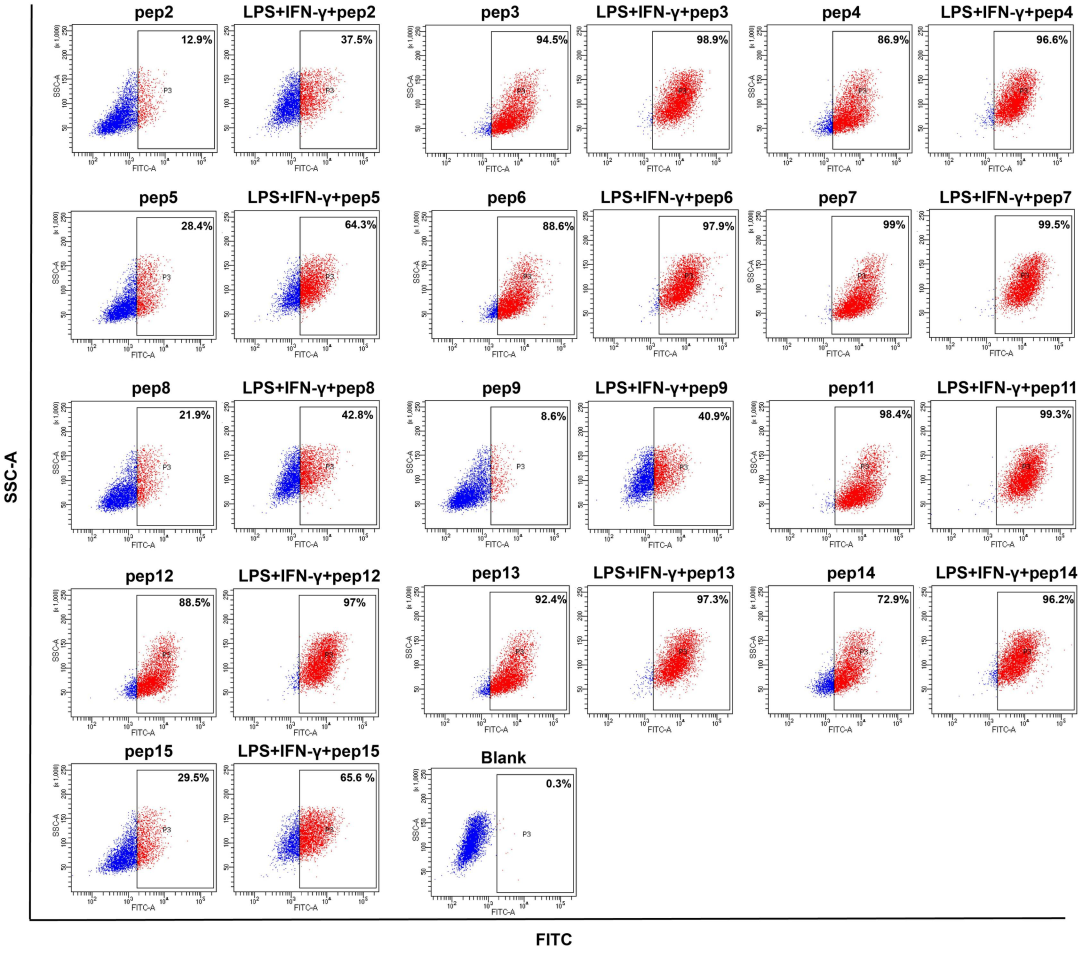

Figure S2 The targeting of polypeptide sequences to M1-polarized RAW264.7 cells by flow cytometry (n=3).

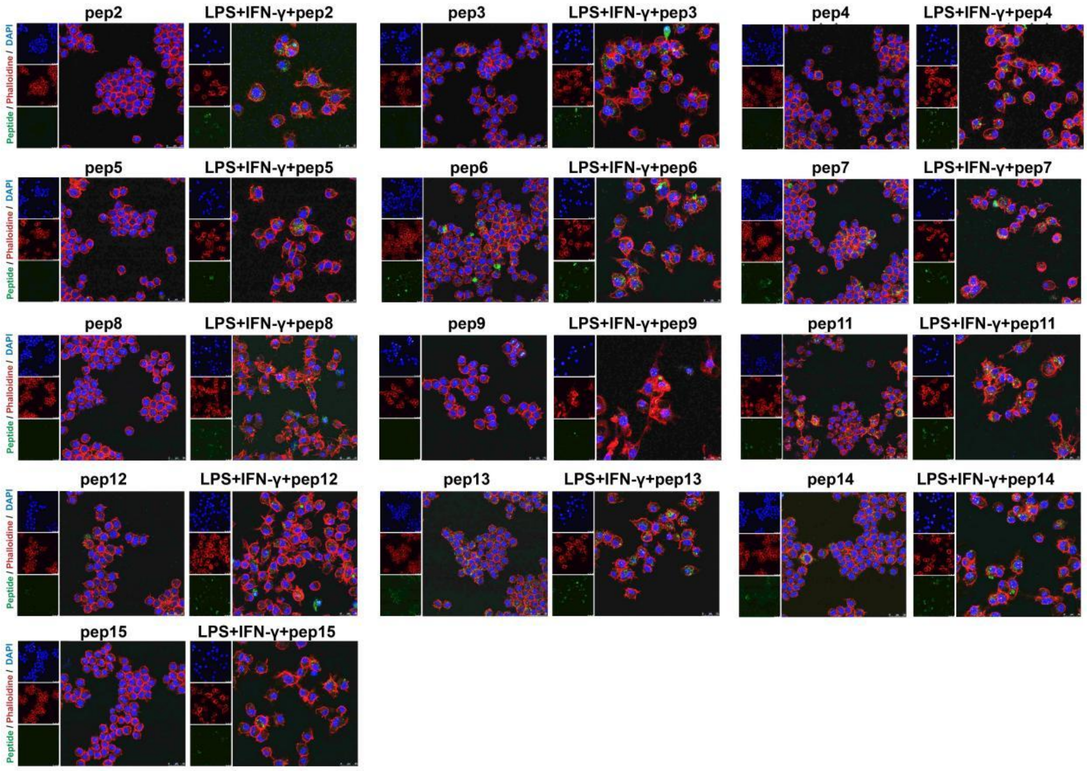

Figure S3 The targeting of polypeptide sequences to M1-polarized RAW264.7 cells by laser confocal imaging (n=3).

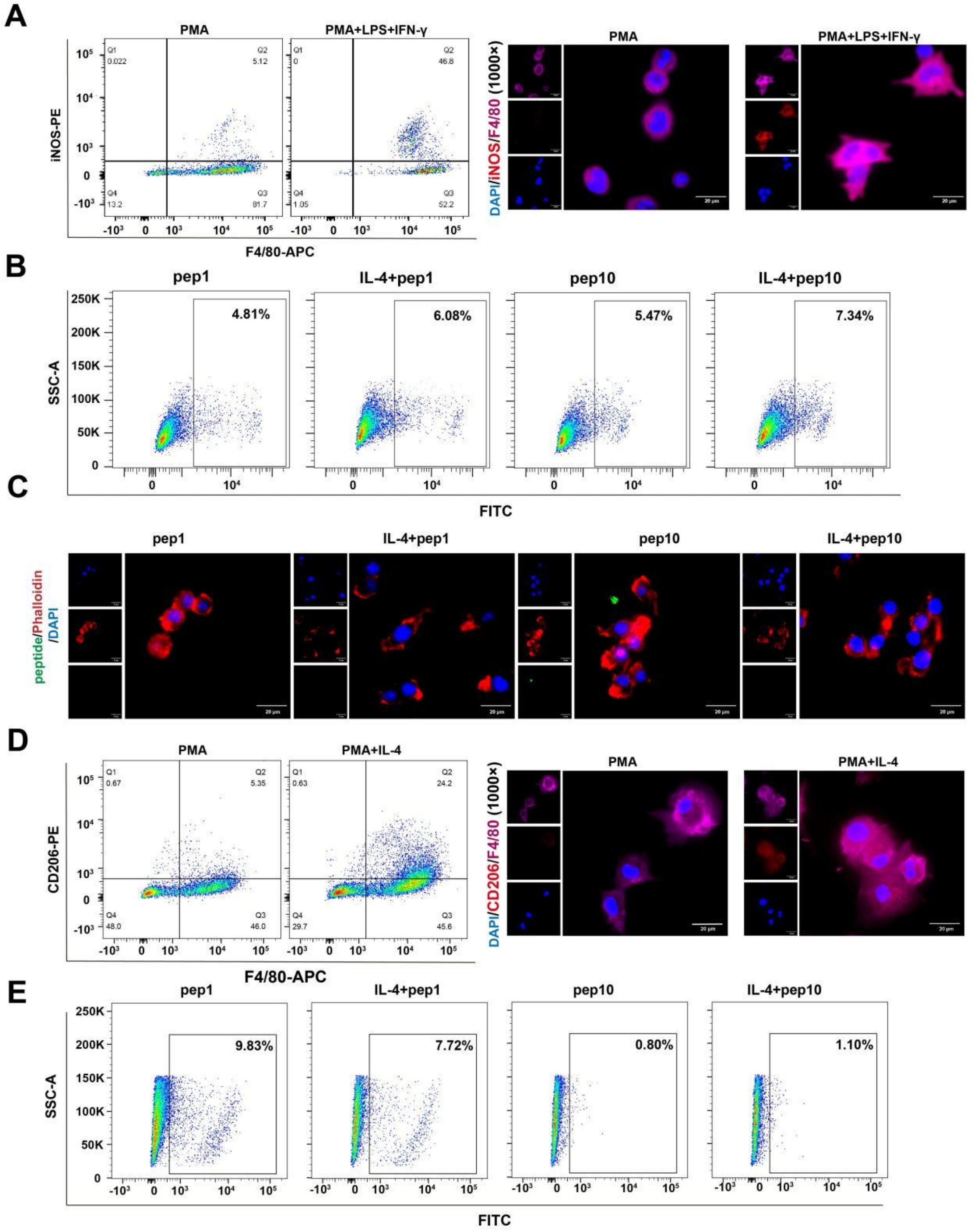

Figure S4 The targeting ability of M1pep towards M2 macrophages.

(A) M1-polarized THP-1 cells were identified by flow cytometrys and immunofluorescence (n=3). (B-C) Flow cytometry and laser confocal imaging for detecting the targeting of peptide sequences to M2-polarized RAW264.7 cells. (D). M2-polarized THP-1 cells were identified by flow cytometrys and immunofluorescence (n=3). (E) Flow cytometry for detecting the targeting of peptide sequences to M2-polarized THP-1 cells.

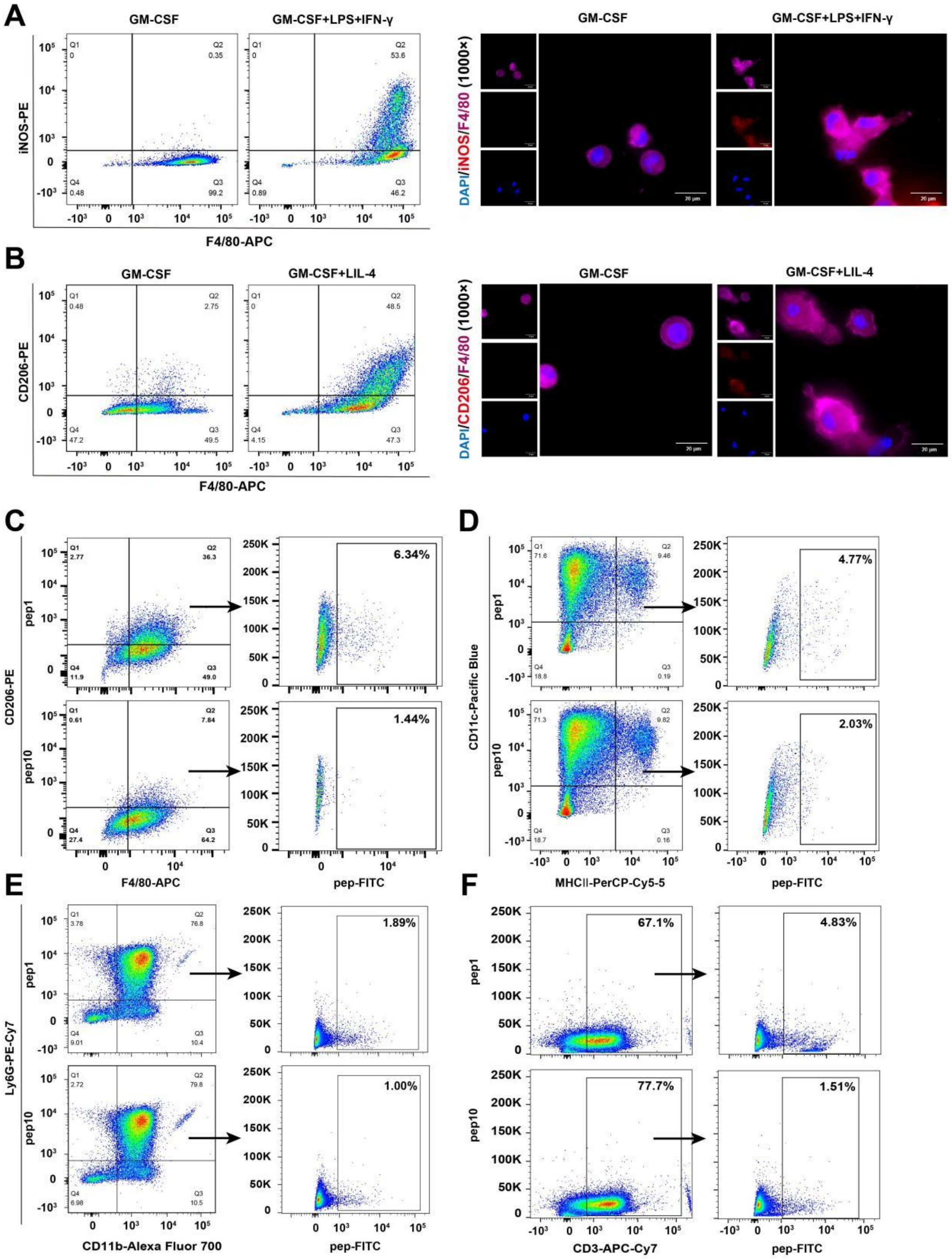

Figure S5 The targeting ability of M1pep towards primary immune cells of mice. (A) M1-polarized BMDM were identified by flow cytometry and immunofluorescence (n=3). (B) M2-polarized BMDM were identified by flow cytometrys and immunofluorescence (n=3). (C) Flow cytometry detection of the targeting ability of M1pep to M2-polarized BMDM. (D). Flow cytometry-based identification of mouse DC and the targeted detection of M1pep on mouse DC. (E) Flow cytometry-based identification of mouse neutrophils and the targeted detection

of M1pep on mouse neutrophils. (F) Flow cytometry-based identification of mouse T cell and the targeted detection of M1pep on mouse T cell.

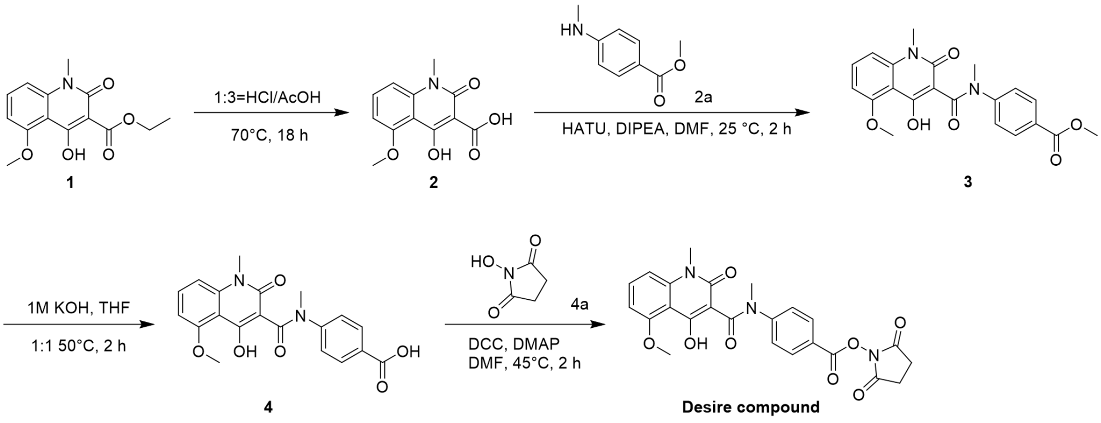

Figure S6 Structural transformation diagram of Tasq.

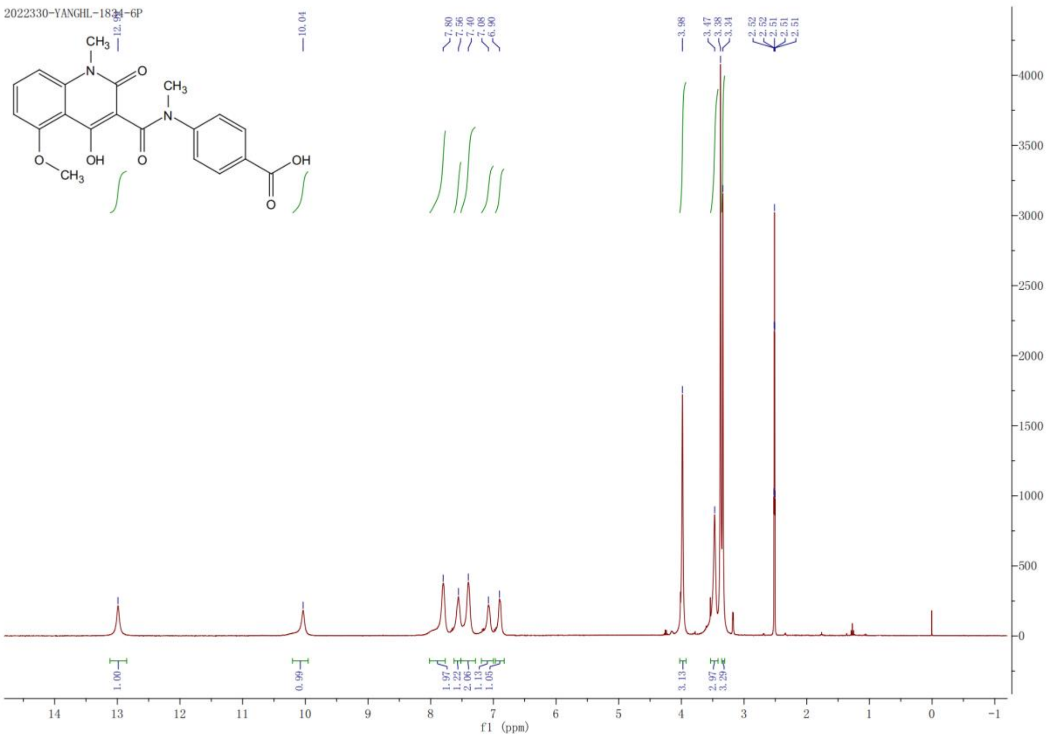

Figure S7 HNMR of the target product after structural modification.

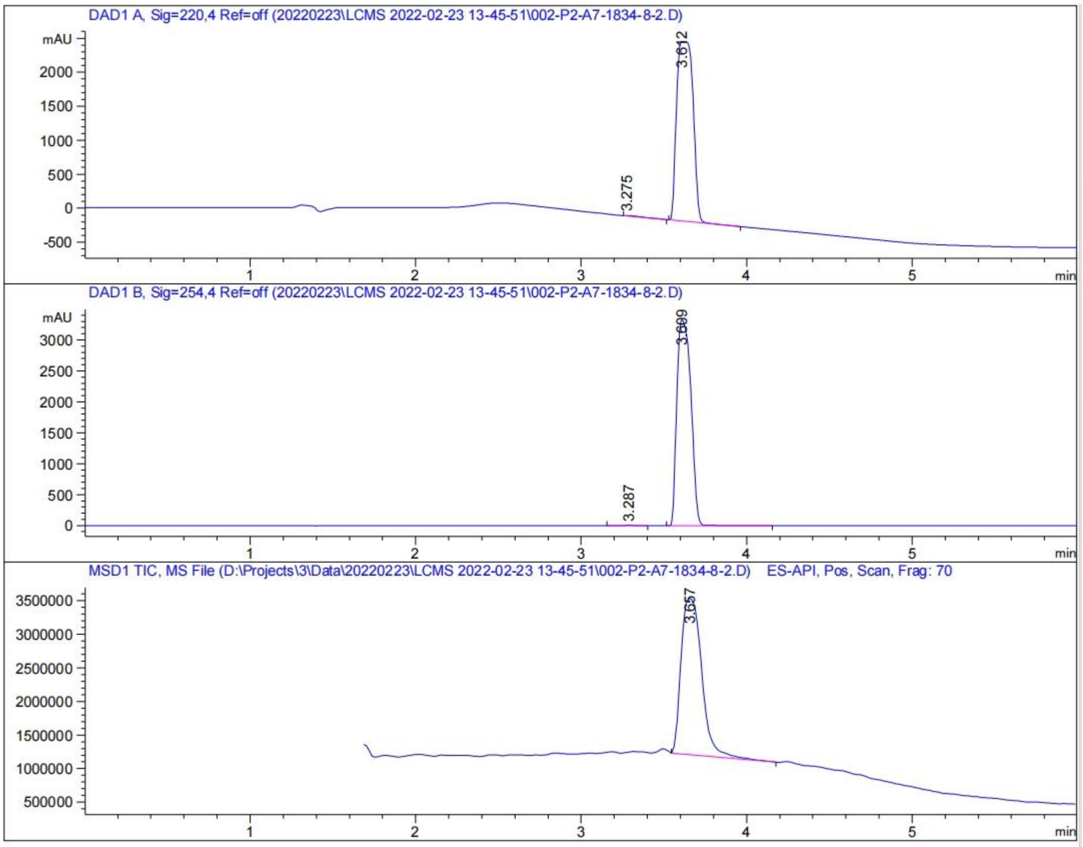

Figure S8 HPLC of the target product after structural modification.

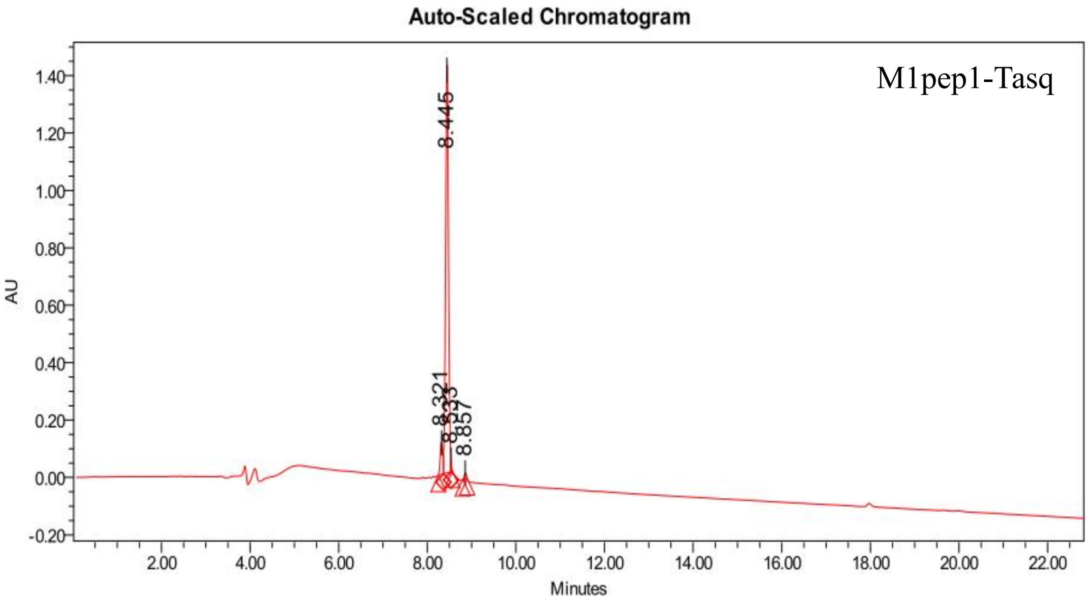

Peak Results

	RT	Area	Height	% Area
1	8.321	458715	110034	6.55
2	8.445	6440841	1406283	92.00
3	8.533	32105	39715	0.46
4	8.857	68983	20418	0.99

Figure S9 HNMR of the M1pep1-Tasq after synthesis.

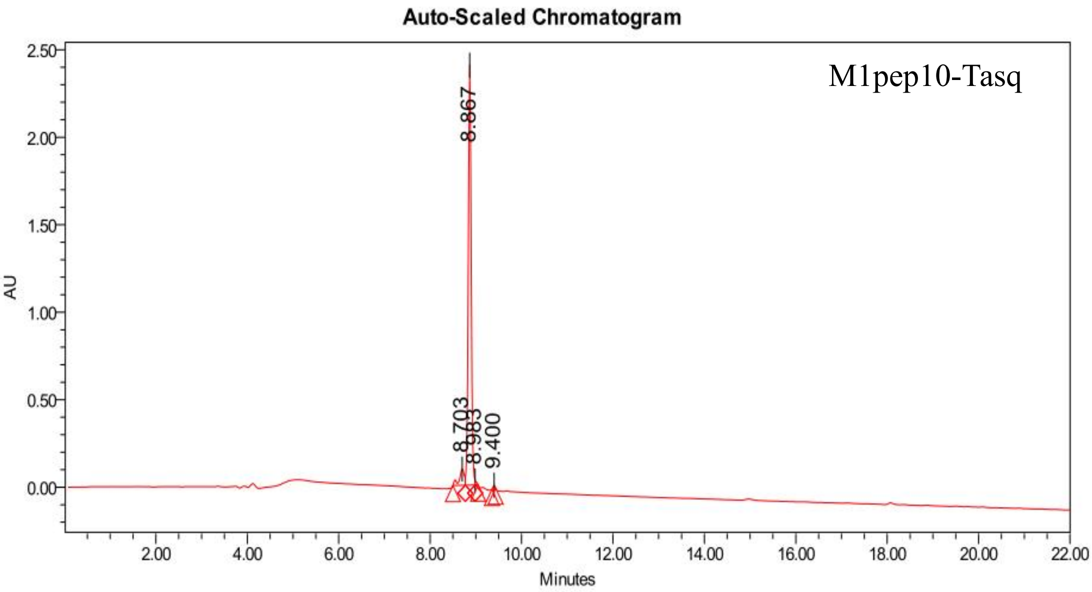

Peak Results

	RT	Area	Height	% Area
1	8.703	677146	92810	5.80
2	8.867	10881657	2355274	93.23
3	8.983	58776	24642	0.50
4	9.400	54705	18897	0.47

Figure S10 HNMR of the M1pep10-Tasq after synthesis.

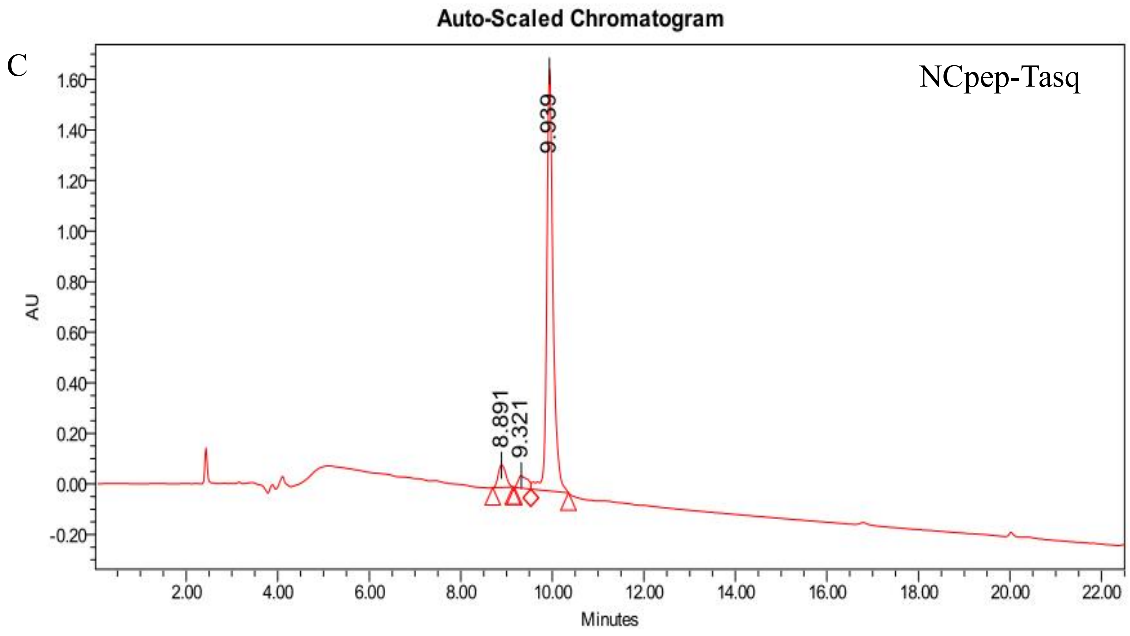

Peak Results

	RT	Area	Height	% Area
1	8.891	1023720	89018	5.69
2	9.321	715112	49239	3.98
3	9.939	16248148	1674673	90.33

Figure S11 HNMR of the NCpep-Tasq after synthesis.

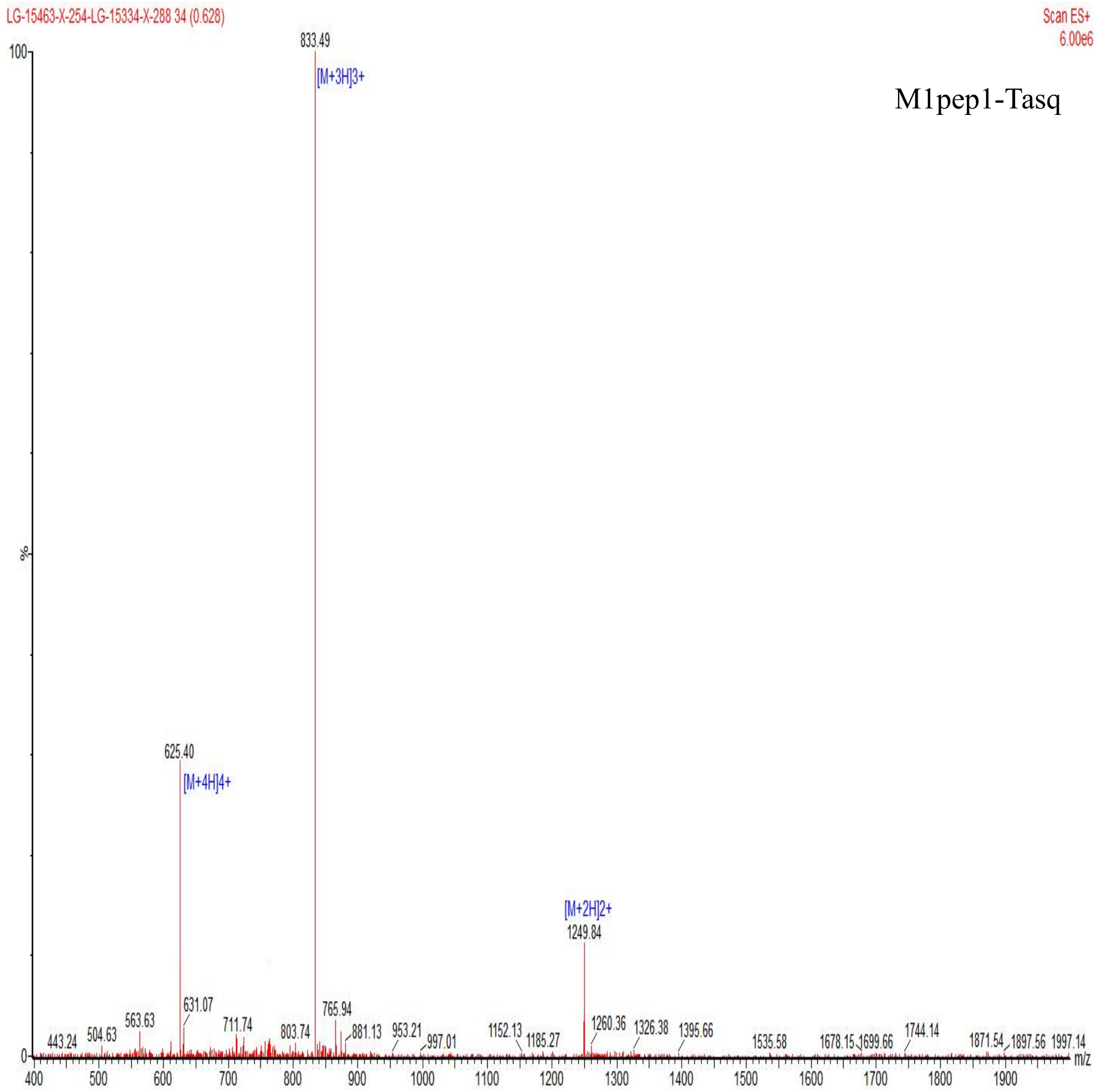

Figure S12 HPLC of the M1pep1-Tasq after synthesis.

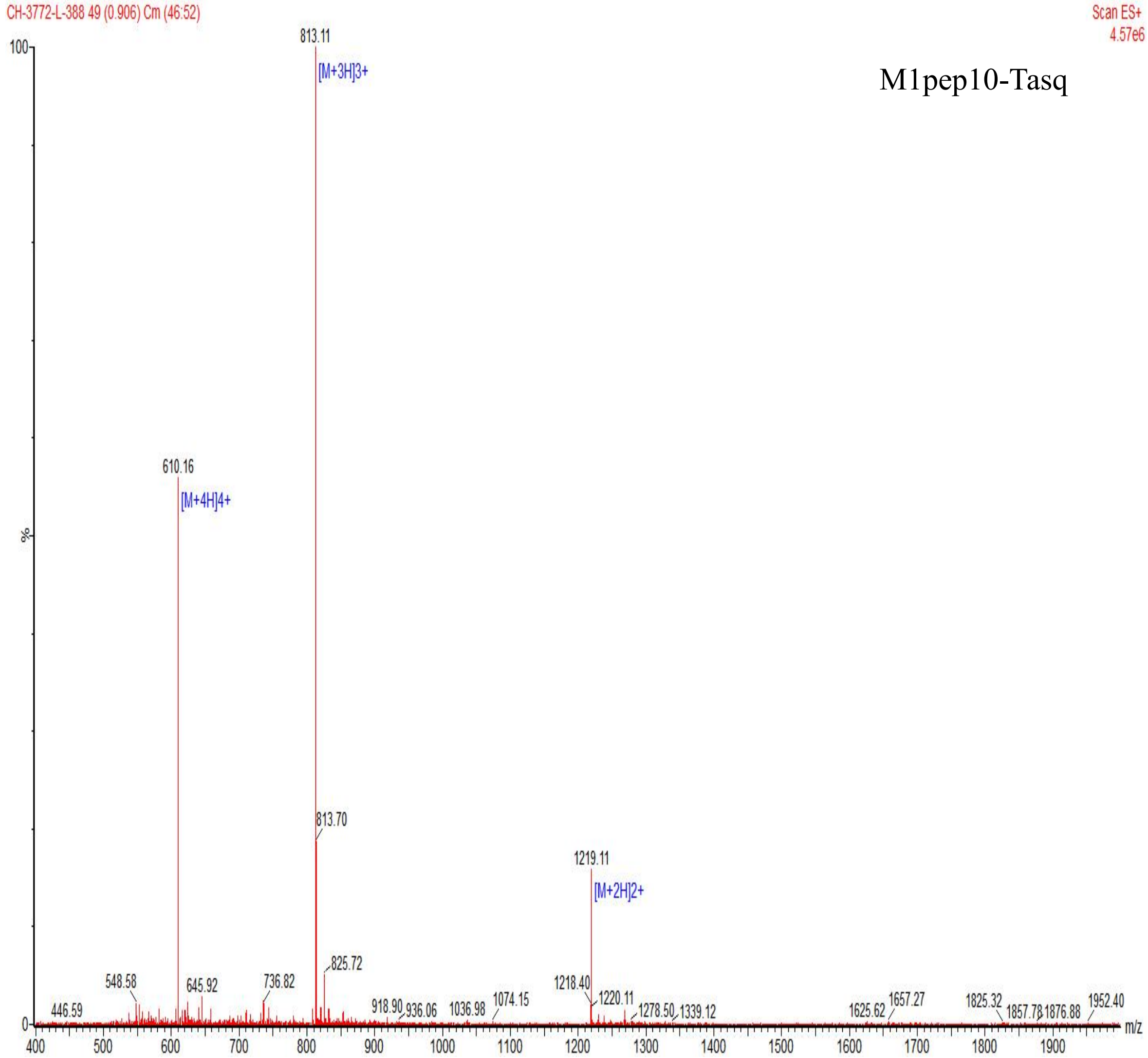

Figure S13 HPLC of the M1pep10-Tasq after synthesis.

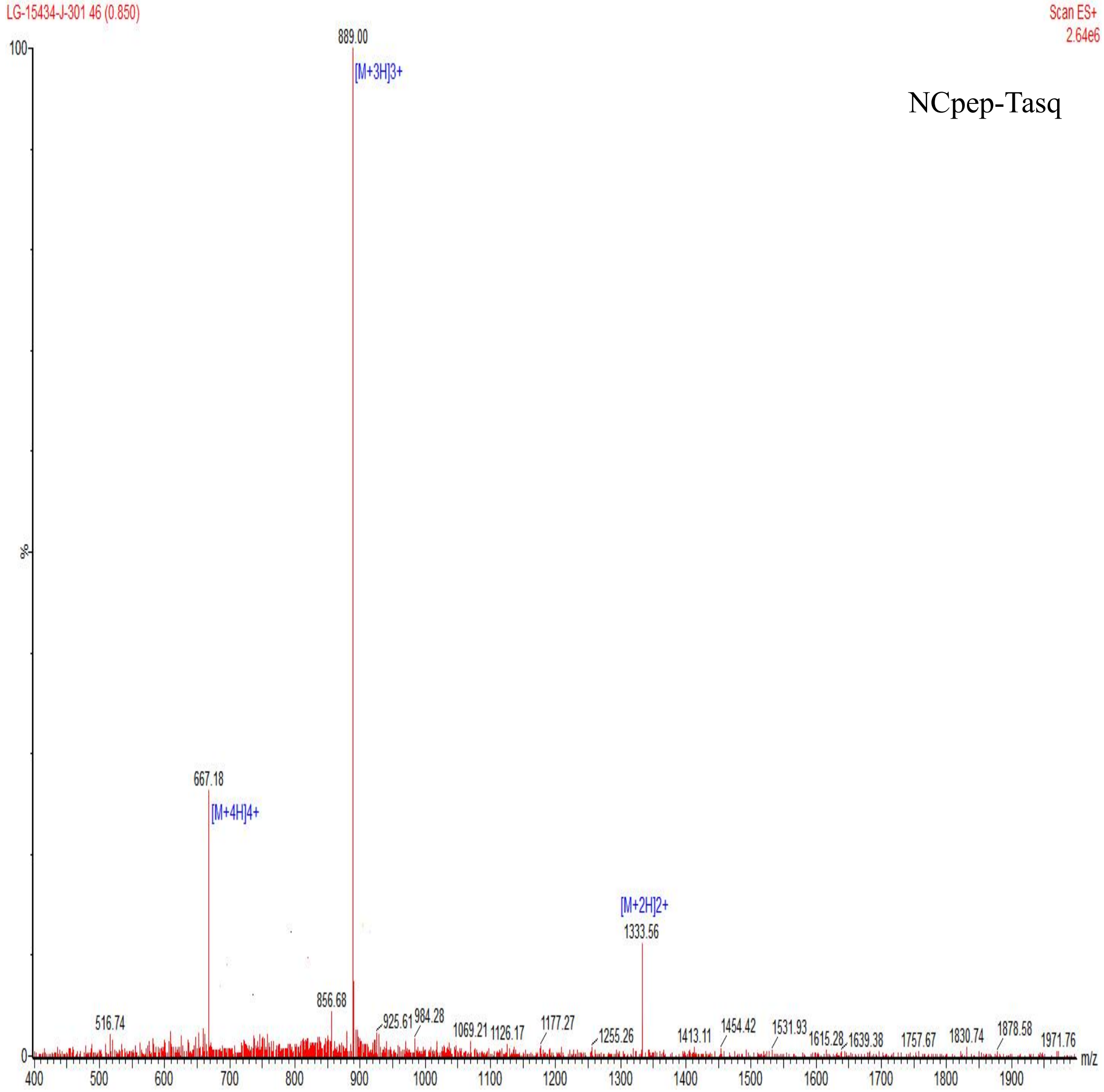

Figure S14 HPLC of the NCpep-Tasq after synthesis.

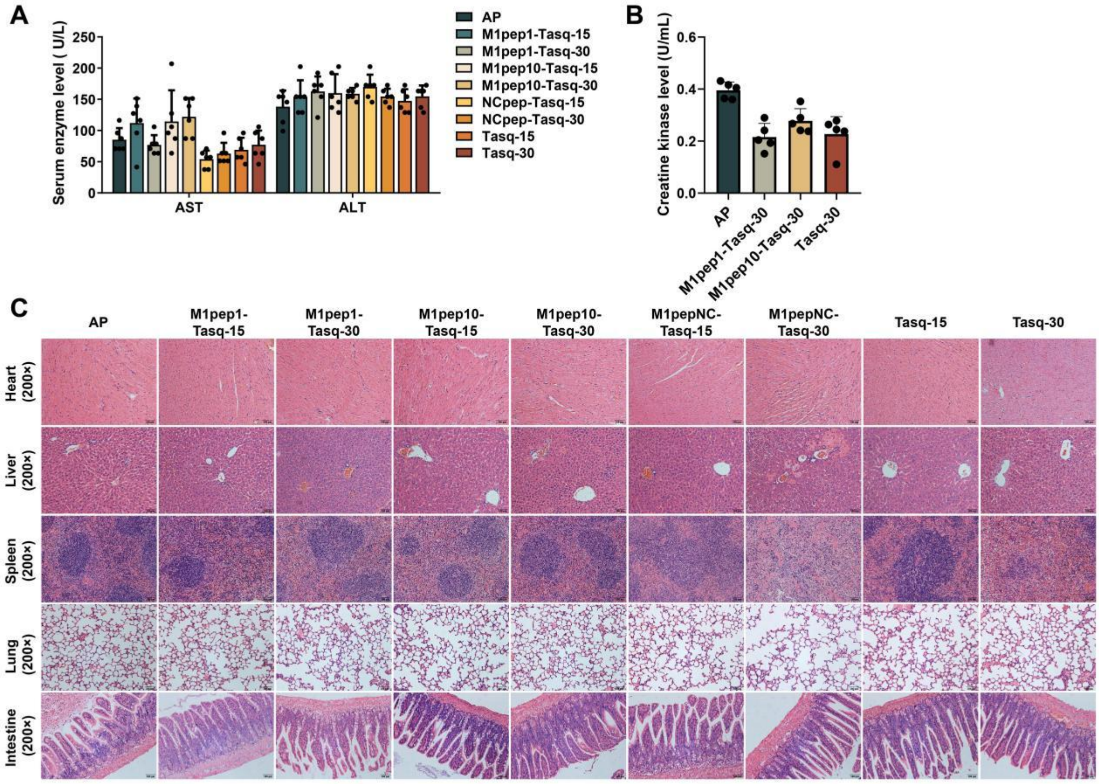

Figure S15 M1pep-Tasq reduces the toxicity of Tasq.

(A) The serum levels of liver function markers, ALT and AST (n=6). (B) The serum levels of heart and skeletal muscle function markers, CK (n=5). (C) HE staining of mouse heart, liver, spleen, lung, intestine. Data were expressed as mean ± SD.

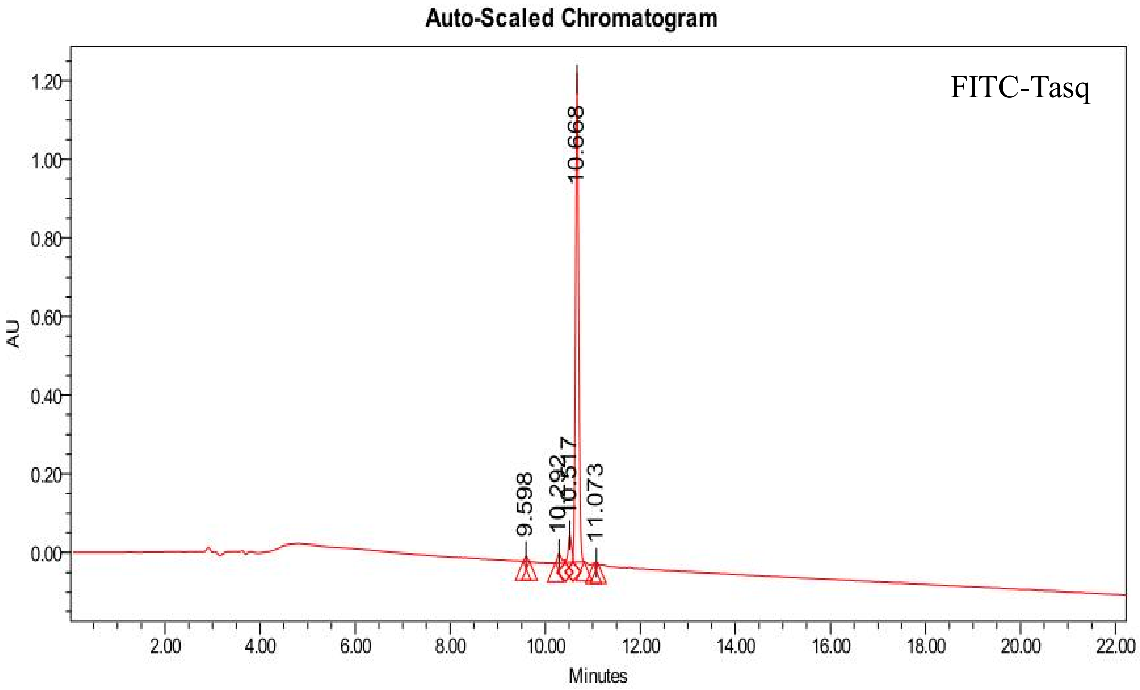

Peak Results

	RT	Area	Height	% Area
1	9.598	52659	13003	0.86
2	10.292	133400	23774	2.19
3	10.517	347754	68279	5.70
4	10.668	5551033	1218331	91.00
5	11.073	14943	4465	0.24

Figure S16 HNMR of the FITC-Tasq after synthesis.

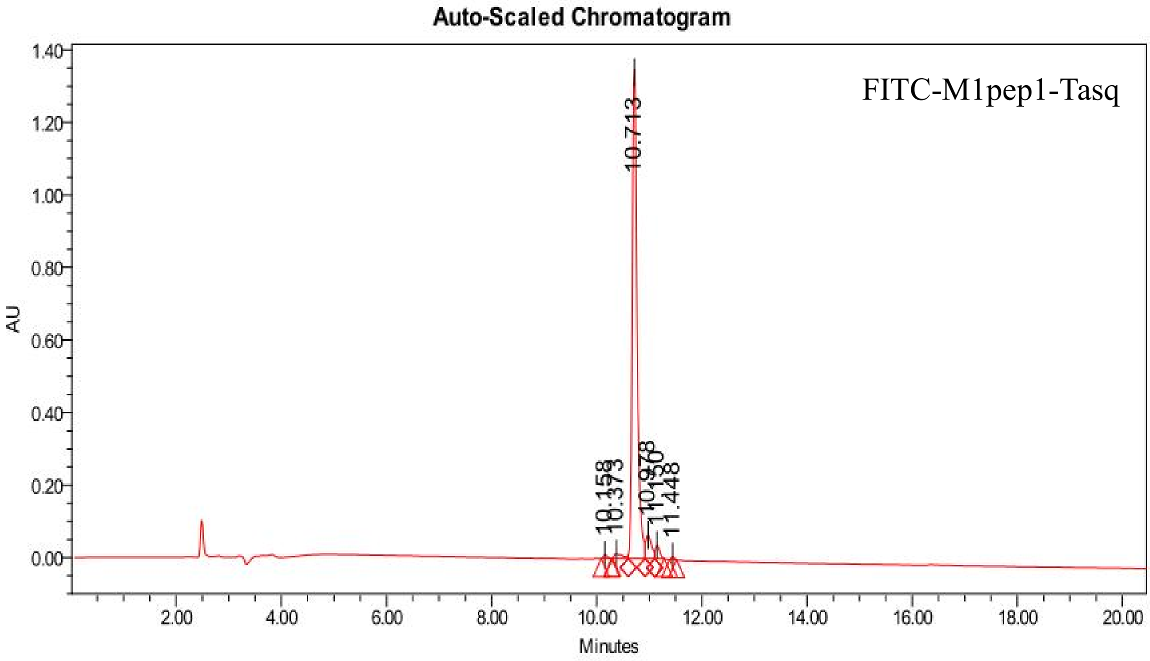

Peak Results

	RT	Area	Height	% Area
1	10.158	56773	10658	0.58
2	10.373	142920	13631	1.47
3	10.713	8784790	1345836	90.30
4	10.978	498776	65456	5.13
5	11.15[illegible]	211841	37344	2.18
6	11.44[illegible]	33210	7953	0.34

Figure S17 HNMR of the FITC- M1pep1-Tasq after synthesis.

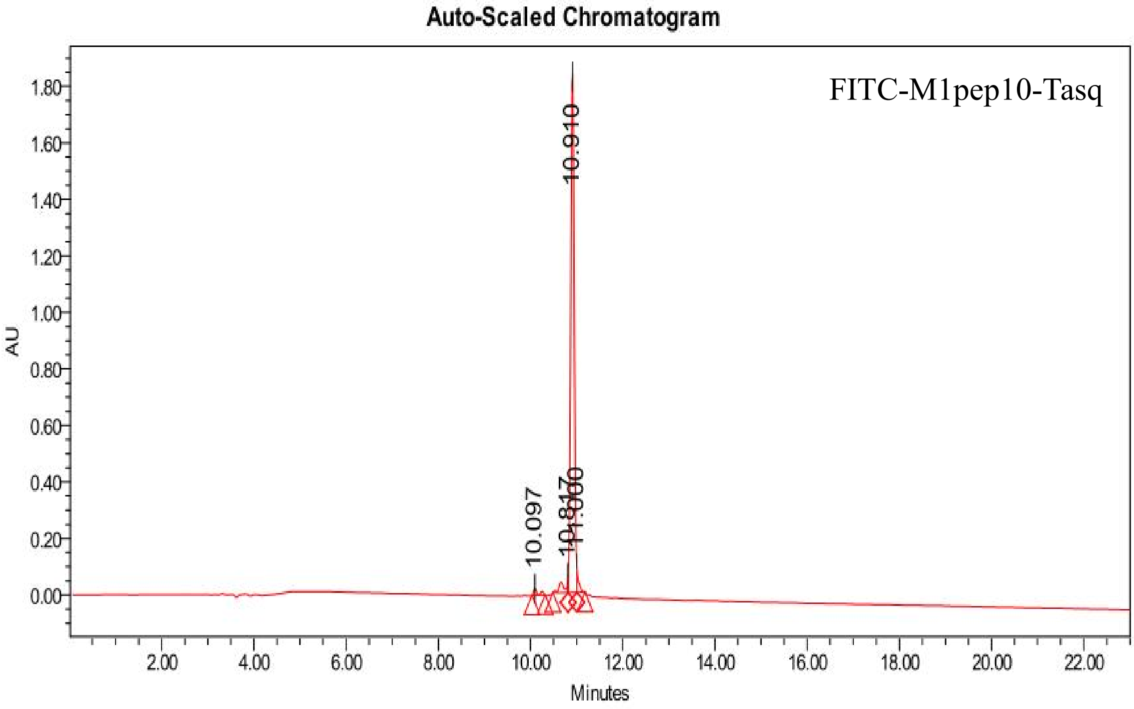

Peak Results

	RT	Area	Height	% Area
1	10.097	148842	23056	1.49
2	10.817	347265	52189	3.47
3	10.910	9270724	1841900	92.76
4	11.000	227903	83070	2.28

Figure S18 HNMR of the FITC- M1pep10-Tasq after synthesis.

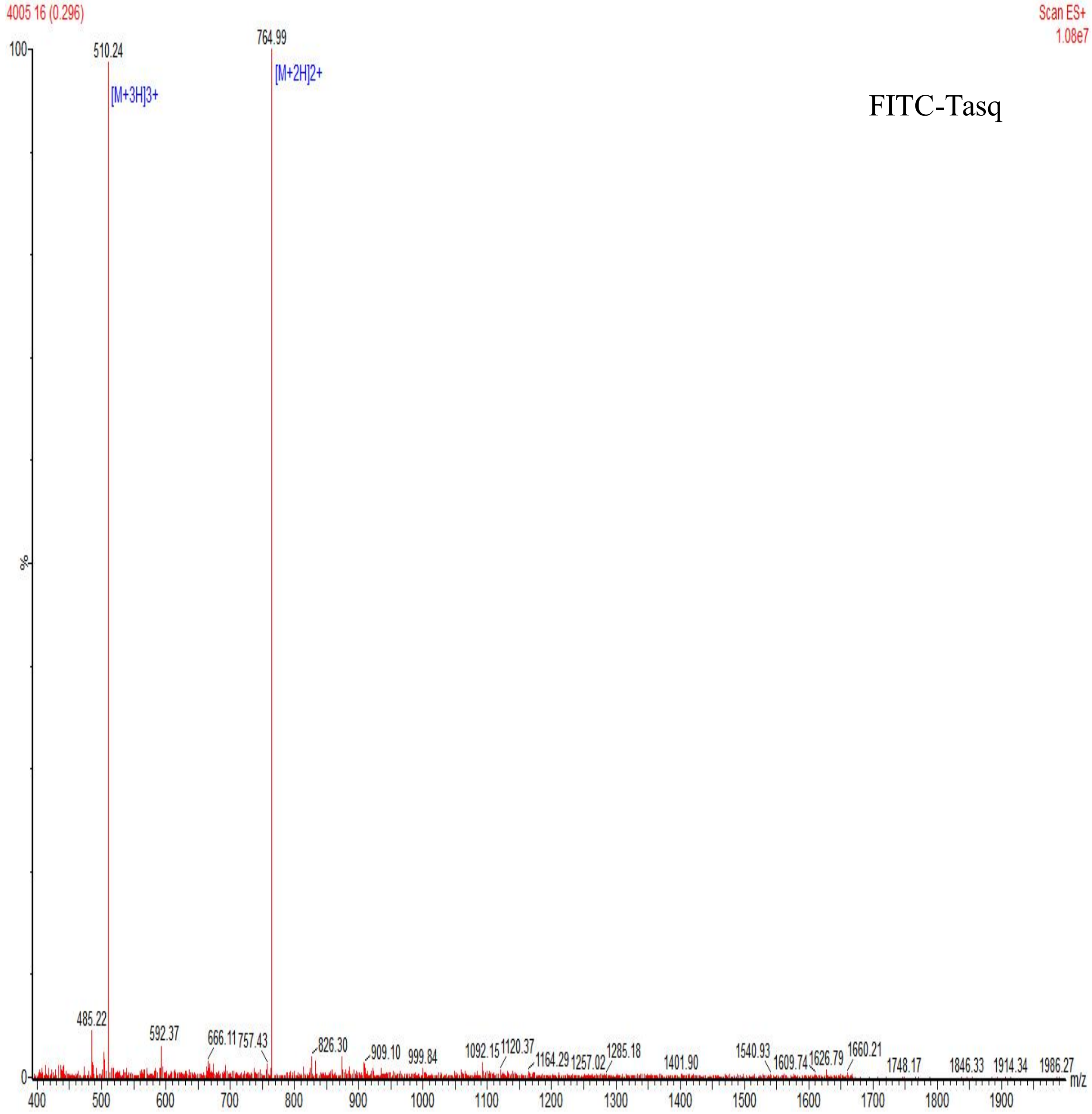

Figure S19 HPLC of the FITC-Tasq after synthesis.

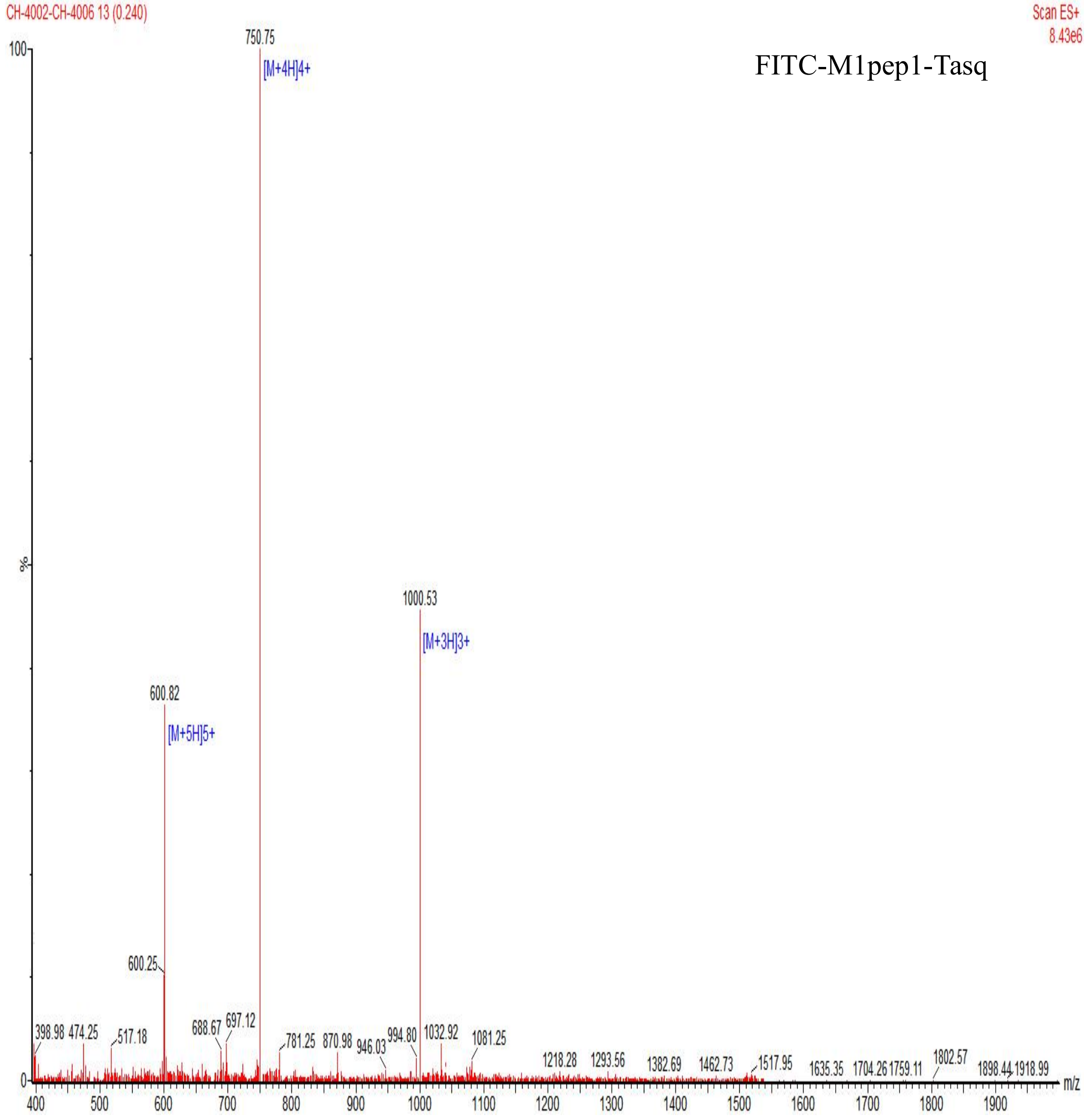

Figure S20 HPLC of the FITC-M1pep1-Tasq after synthesis.

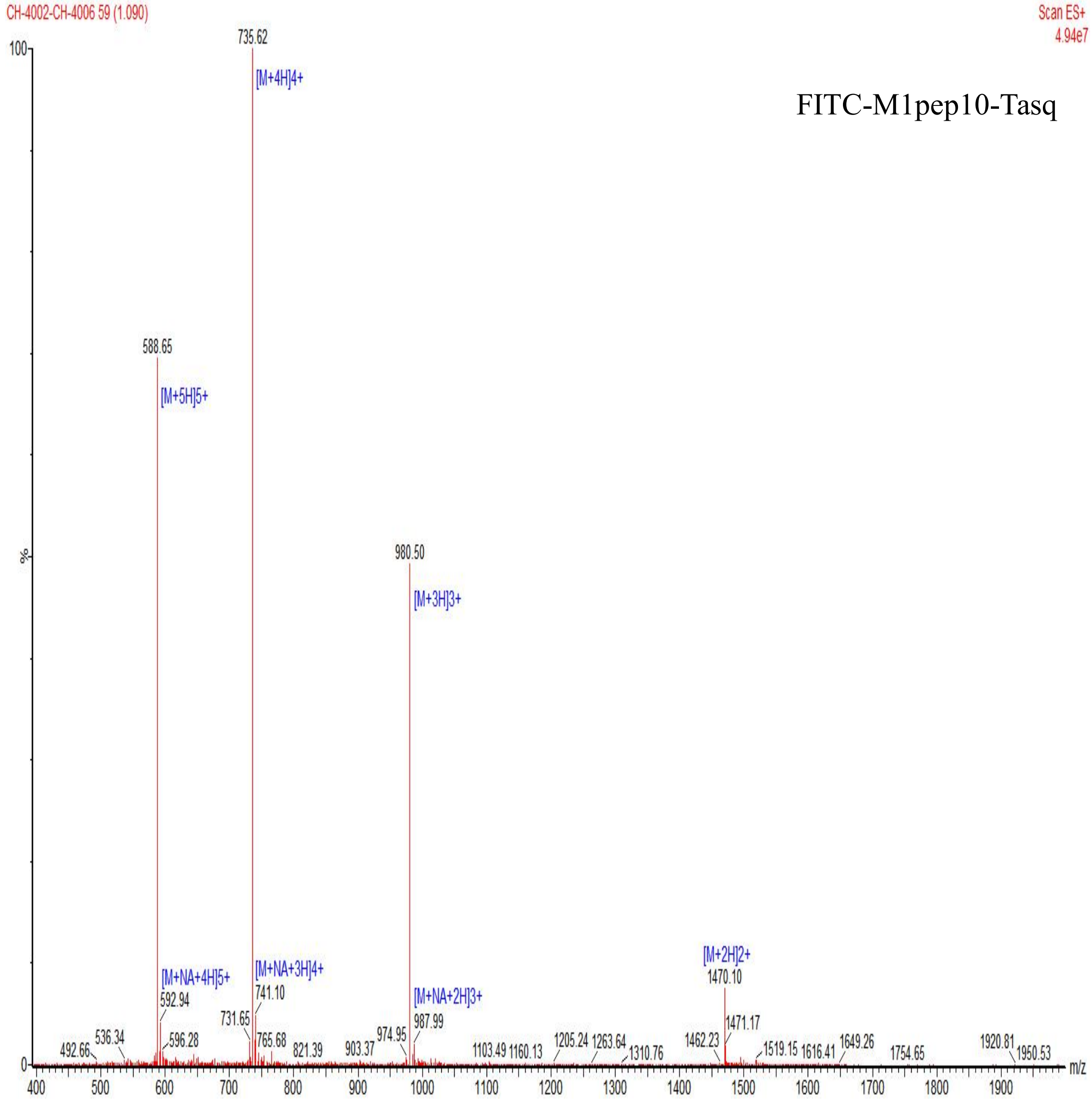

Figure S21 HPLC of the FITC-M1pep10-Tasq after synthesis.

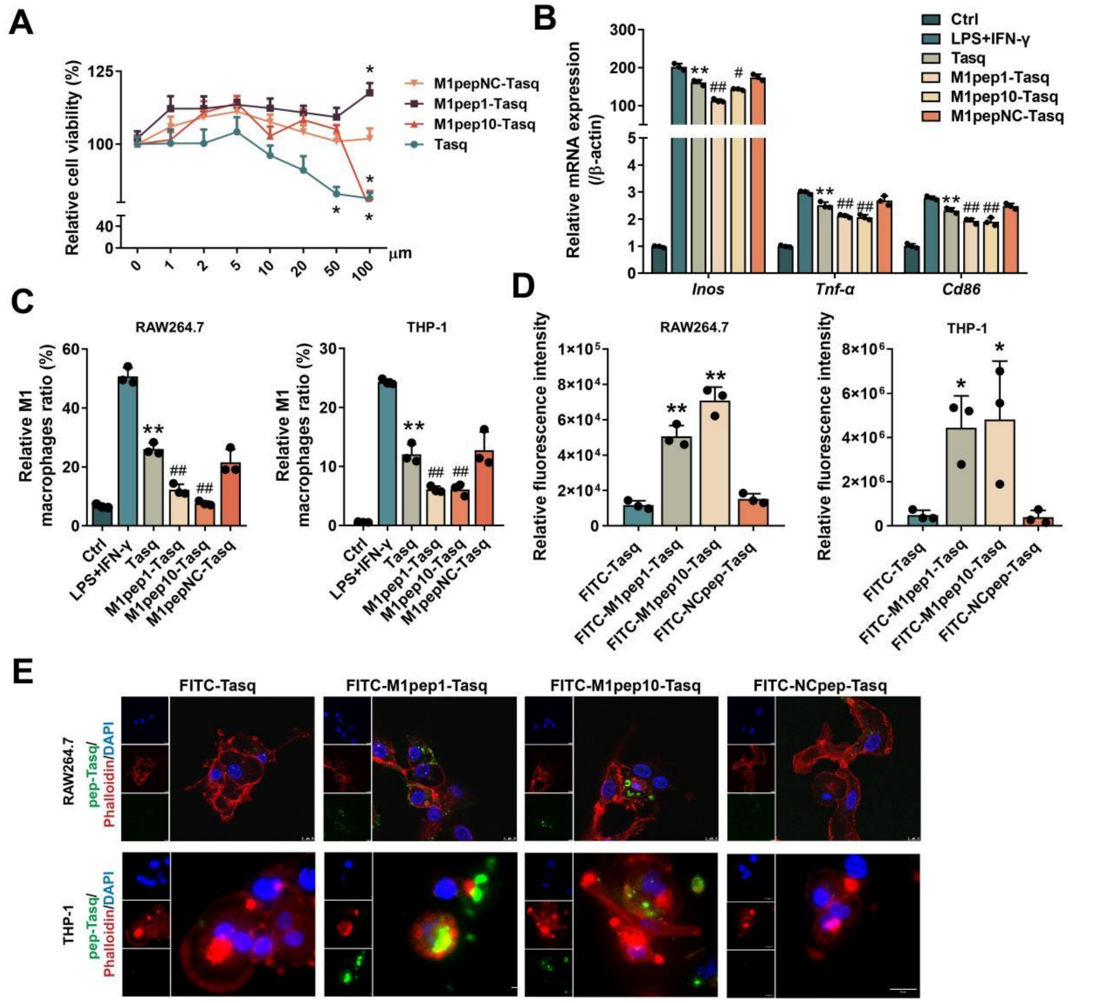

Figure S22 M1pep-Tasq enhances the efficacy of Tasq by enhancing the targeting ability of Tasq.

(A) The toxicity of Tasq, M1pep1-Tasq, M1pep10-Tasq and NCpep-Tasq to RAW264.7 cells was detected by CCK8 assay. Data were expressed as mean ±SD, **P* < 0.05 vs. 0 μM. (B) The mRNA expression of M1-like macrophage polarization markers in RAW264.7 cell (n=3). (C) Flow cytometric analysis of M1-like macrophages in RAW264.7 cell and THP-1 cell (n=3). Data were expressed as mean ±SD, ***P* < 0.01 vs. LPS+IFN-γ $^{\#}P$ < 0.05, $^{\#\#}P$ < 0.01 vs. Tasq. (D-E) Immunofluorescence detection of M1-like macrophage targeting of FITC-Tasq, FITC-M1pep1-Tasq, FITC-M1pep10-Tasq and FITC-NCpep-Tasq.

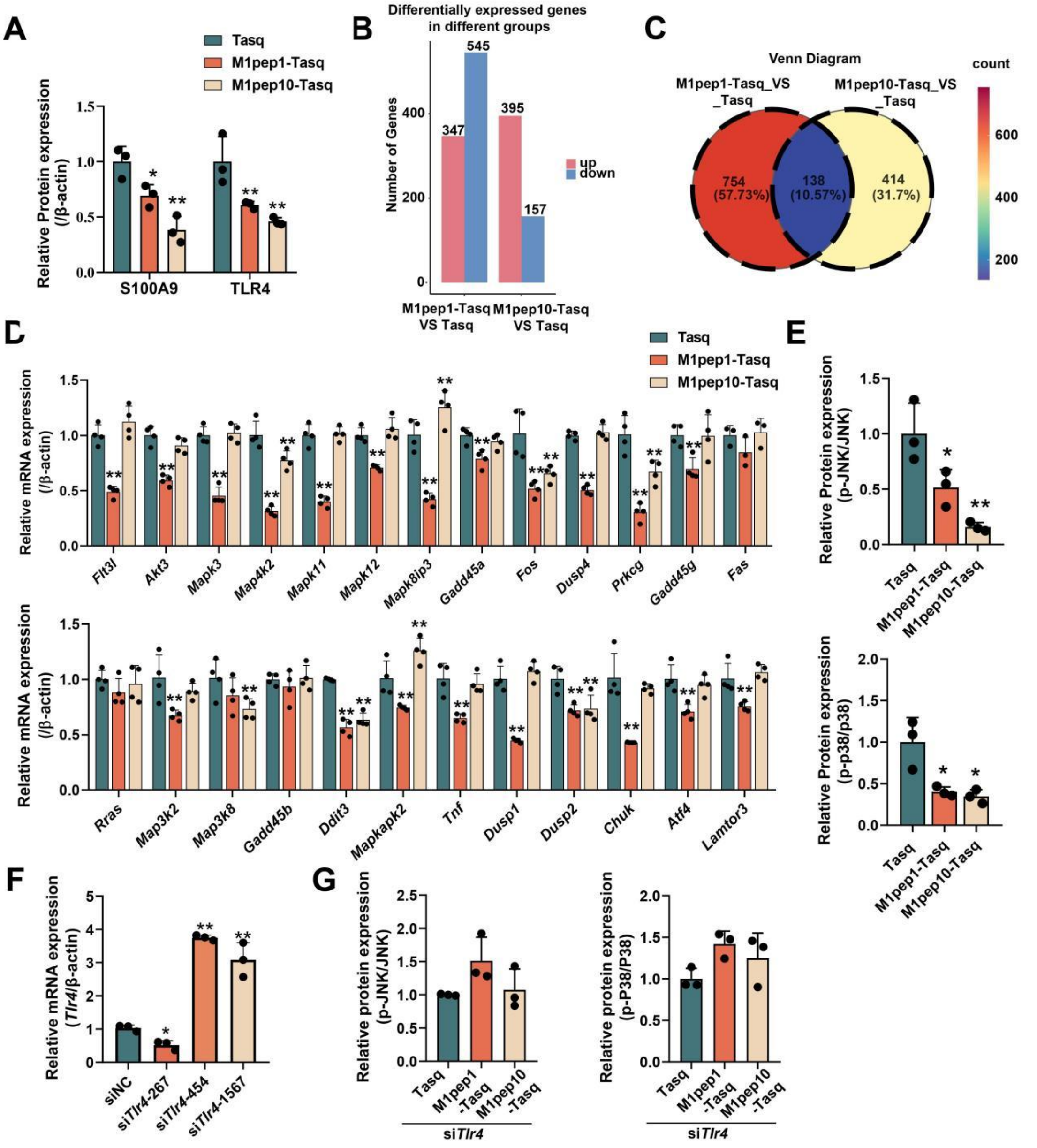

Figure S23 M1pep-Tasq inhibits macrophages M1 polarization by inhibiting S100A9-TLR4-MAPK pathway.

(A) Analysis of S100A9 and TLR4 protein expression (n=3). (B) Statistical graph of the genetic quantity of pancreatic organs in mice with different processing groups. (C) Venn diagram of the number of genes in the pancreas. (D) *In vitro* validation of relevant differential genes by qPCR (n=4). (E) Analysis of JNK, p-JNK, P38 and p-P38 protein expression (n=3). (F) Verification of *Tlr4* knockdown efficiency in RAW264.7 cells (n=3). (G) Analysis of JNK, p-JNK, P38 and p-P38 protein expression (n=3). Data were expressed as mean±SD, *$P < 0.05$, **$P < 0.01$ vs. Tasq or si*Tlr4*+Tasq.

Table S1. The primers used in this study.

Gene	Primers (5'-3')
β-actin	Forward: CATCCGTAAAGACCTCTATGCCAAC
	Reverse: ATGGAGCCACCGATCCACA
inos	Forward: ACTCAGCCAAGCCCTCACCTAC
	Reverse: TCCAATCTCTGCCTATCCGTCTCG
tnf-α	Forward: ATGTCTCAGCCTCTTCTCATTC
	Reverse: GCTTGTCACTCGAATTTTGAGA
cd86	Forward: ACGGAGTCAATGAAGATTTCCT
	Reverse: GATTCGGCTTCTTGTGACATAC
il-1β	Forward: AAATCTCGCAGCAGCACATCAA
	Reverse: CCACGGGAAAGACACAGGTAGC
il-6	Forward: CCTTCTTGGGACTGATGATGCTG
	Reverse: TTGGGAGTGGTATCCTCTGTGA
il-18	Forward: GACAGCCTGTGTTCGAGGATATG
	Reverse: TGTTCTTACAGGAGAGGGTAGAC
flt3l	Forward: CGCTGGATAGAGCAACTGAAGAC
	Reverse: TGTTGGTCTGGACGAATCGCAG
mapk3	Forward: GGCTTTCTGACGGAGTATGTGG
	Reverse: GTTGGAGAGCATCTCAGCCAGA
map4k2	Forward: GGCTACTCTGAAGCAACAGGAG
	Reverse: GCAGCCATTGAAGACCTTGGAG
akt3	Forward: GAGATGGATGCGTCTACAACCC
	Reverse: TCCACTTGCCTTCTCTCGAACC
mapk11	Forward: CAGAAGGACCTCAGCAGTGTCT
	Reverse: GTACTGGCTGAAGTATGCGTGG
mapk8ip3	Forward: CCAGTACAAGGAACGCCTCATG
	Reverse: GGCTAGATGAGGAGCTGAAGAG
dusp4	Forward: CTCCTGGTTCATGGAAGCCATC
	Reverse: GACGAACTCAAAAGCCTCCTCC
tnf	Forward: GGTGCCTATGTCTCAGCCTCTT
	Reverse: GCCATAGAACTGATGAGAGGGAG
dusp1	Forward: CAACCACAAGGCAGACATCAGC
	Reverse: GTAAGCAAGGCAGATGGTGGCT
prkcg	Forward: ACGCAGCTTCACTCCACCTTTC
	Reverse: TGGCGATTTCCGCAGCGTAGAA

map3k2	Forward: CCCAGAGTATGACGACAGTCGA<br>Reverse: GGTAGACCCTACCAAAAGCTCC
mapkapk2	Forward: GCACTCGATCAACATTGCTCACC<br>Reverse: TGTGACTGGTGGTTTCCTTGGC
atf4	Forward: AACCTCATGGGTTCTCCAGCGA<br>Reverse: CTCCAACATCCAATCTGTCCCG
Lamtor3	Forward: AGACCTGGCTTCCTATCCACGT<br>Reverse: AACTCACCACCAGAGGCAAACG
chuk	Forward: TCGGAAACCAGCCTCTCAGTGT<br>Reverse: CTTCTGGATGCAAATGGTCCTTC
gadd45b	Forward: GGAGACATTGGGCACAACCGAA<br>Reverse: CTGCTCTCTTCACAGTAACTGGC
ddit3	Forward: GGAGGTCCTGTCCTCAGATGAA<br>Reverse: GCTCCTCTGTCAGCCAAGCTAG
fos	Forward: GGGAATGGTGAAGACCGTGTCA<br>Reverse: GCAGCCATCTTATTCCGTTCCC
map3k8	Forward: CTTTGAACGGAAGAGGCTGCTG<br>Reverse: GAACGCTGTCTCCTGAGCACTT
rras	Forward: GGCAAGCTCTTCACACAGATCC<br>Reverse: GGAAGCACTGAAAGAGGAGGCT
Dusp2	Forward: AGATGGTGGAGATAAGTGCCTGG<br>Reverse: AGATGGTGGCTGAGCGAGAGAT
gadd45g	Forward: TCTACGAGTCCGCCAAAGTCCT<br>Reverse: CTCACAGCAGAACGCCTGAATC
fas	Forward: CTGCGATTCTCCTGGCTGTGAA<br>Reverse: CAACAACCATAGGCGATTTCTGG
gadd45a	Forward: CCTGGAGGAAGTGCTCAGCAAG<br>Reverse: GTCGTCTTCGTCAGCAGCCAG
mapk12	Forward: GGCTACTGGATGTGTTCACACC<br>Reverse: CTGGATTCTGTCTTCACTCAGGG

Table S2. Polypeptide positive sequence

No.	**Amino acid sequence**
1	FSDDCYDCRIPR
2	AALSYSI
3	DLWFPLP
4	DSWFHTISEAGW
5	SWKHDHFTAFRN
6	NLWFPLEWPVPA
7	DLWFPWN
8	GTYYNHSIPWNI
9	VHLDNVK
10	VHAVPIRTIYYP
11	DSWFPLDSFDEL
12	DPWFHWELSSMG
13	DLWFPIS
14	SWLDLIQNSLSY
15	TASTSLN
